# Total Synthesis and Metabolic Stability of Hispidulin and Its *d*-Labelled Derivative

**DOI:** 10.3390/molecules22111897

**Published:** 2017-11-04

**Authors:** Liang-Chieh Chen, Kai-Cheng Hsu, Lih-Chu Chiou, Hui-Ju Tseng, Wei-Jan Huang

**Affiliations:** 1 Graduate Institute of Pharmacognosy, College of Pharmacy, Taipei Medical University, Taipei 110, Taiwan; pigjay15@gmail.com (L.-C.C.); d343105007@tmu.edu.tw (H.-J.T.); 2 School of Pharmacy, College of Pharmacy, Taipei Medical University, Taipei 110, Taiwan; 3 Program for Cancer Biology and Drug Discovery, College of Medical Science and Technology, Taipei Medical University, Taipei 110, Taiwan; piki@tmu.edu.tw; 4 Graduate Institute of Brain and Mind Sciences, College of Medicine, National Taiwan University, Taipei 110, Taiwan; lcchiou@ntu.edu.tw; 5 Graduate Institute of Pharmacology, College of Medicine, National Taiwan University, Taipei 100, Taiwan; 6 Program in Biotechnology Research and Development, College of Pharmacy, Taipei Medical University, Taipei 110, Taiwan; 7 Program for the Clinical Drug Discovery from Botanical Herbs, Taipei 110, Taiwan; 8 School of Pharmacy, National Defense Medical Center, Taipei 114, Taiwan

**Keywords:** hispidulin, total synthesis, flavone, microsome stability, deuterium-labelled compound

## Abstract

Hispidulin is a naturally occurring flavone known to have various Central nervous system (CNS) activities. Proposed synthetic approaches to synthesizing hispidulin have proven unsatisfactory due to their low feasibility and poor overall yields. To solve these problems, this study developed a novel scheme for synthesizing hispidulin, which had an improved overall yield as well as more concise reaction steps compared to previous methods reported. Additionally, using the same synthetic strategy, *d*-labelled hispidulin was synthesized to investigate its metabolic stability against human liver microsome. This work may produce new chemical entities for enriching the library of hispidulin-derived compounds.

## 1. Introduction

Flavonoids, a group of polyphenolic compounds, occur ubiquitously in plants. These compounds have generally been categorized into several classes, including chalcones, flavonols, flavones, isoflavones, flavan-3-ols, neoflavones, flavanones and anthocyanins, based on their chemical structures [1]. In addition to the diverse structures, they also have many biological properties, such as antioxidative [2], anti-inflammatory [3], antimicrobial [4], anticonvulsant [5], antidepressant [6] and anticancer [7] activities. These facts indicate the important role of flavonoid compounds. 

**Figure 1:**
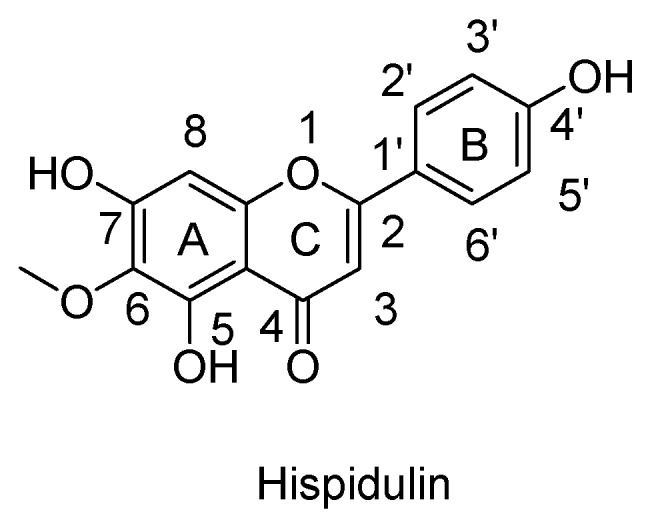
The chemical structure of hispidulin.

Our recent clinical case report described a patient with a refractory chronic motor tic disorder who dramatically improved after taking the leaf juice of a local herb, *Clerodendrum inerme* (L.) Gaertn (*CI*) [8]. The ethanol extract of *CI* leaf alleviated methamphetamine-induced hyperlocomotion (MIH) as a mouse model of motor tic [9]. Using a bioassay-guided purification procedure in this animal model, we further isolated the constituents from *CI* leaf extract to identify a flavone hispidulin (6-methoxy-4′,5,7-trihydroxyflavone) to be the main active ingredient (Figure 1) [9]. Hispidulin significantly attenuated MIH and even in amounts up to 100 mg/kg was incapable of affecting spontaneous locomotor activity or performance in mice [9]. Importantly, hispidulin had no hit on human ether-à-go-go-related gene (hERG) channels, an undesirable target in drug development [10].

Hispidulin is widely distributed in Asteraceae [11,12,13,14] and some of the Lamiaceae [15]. Further assays for screening towards 92 target proteins associated with Central nervous system (CNS) diseases indicated that hispidulin formed strong bonds with GABA_A_ receptors (IC_50_ = 0.73−1.78 μM) and inhibited catecholamine-*O*-methyl-transferase (COMT) (IC_50_ 1.32 μM) [10]. By acting as a positive allosteric modulator (PAM), it enhanced cerebellar α_6_GABA_A_ receptor activity. Studies revealed some C6-substituted flavones are identified to be PAMs of GABA_A_ receptors [16,17]. The results of a structure–activity relationship (SAR) study indicated the C6 substituent in flavones greatly contributes to activity [10].

**Scheme 1:**
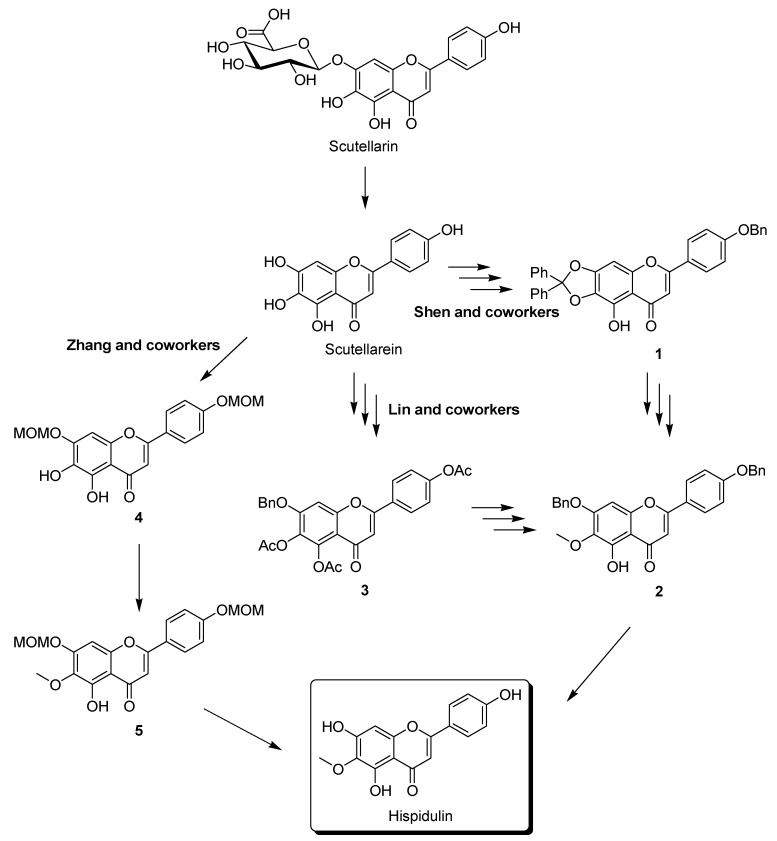
Synthesis of hispidulin starting from scutellarin.

Because of its interesting biological activity, several research groups have developed synthetic approaches to hispidulin [18,19,20,21,22,23]. For example, Shen and coworkers developed a strategy for semisynthesis of hispidulin in seven reaction steps by using a naturally occurring scutellarin (Scheme 1) [22]. Although this method is concise and has an overall yield as high as 10.7% [22], the researchers showed that, upon the large-scale synthesis of compound **1**, the protection of the catechol moiety of scutellarein using dichlorodiphenylmethane at 175 °C failed [20,23]. A seven-step synthesis route developed by Lin and coworkers solved this problem but reduced the overall yield to 7.1% (Scheme 1) [20]. Zhang and coworkers then developed a scheme that only required four reaction steps (Scheme 1). Nonselective MOM (methoxymethyl) protection of scutellarein caused the overall yield of the synthesis of hispidulin to be decreased (6.3%) [23]. Despite the satisfactory overall yield of these strategies, the tedious purification procedure required to isolate scutellarin from plants limits their use for large-scale preparation of hispidulin. 

**Scheme 2:**
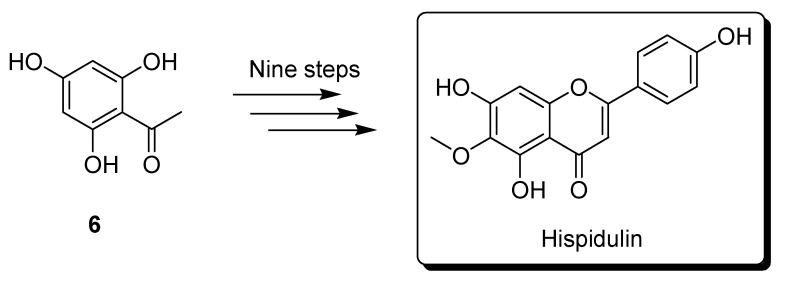
Synthesis of hispidulin by Kavvadias and coworkers.

**Scheme 3:**
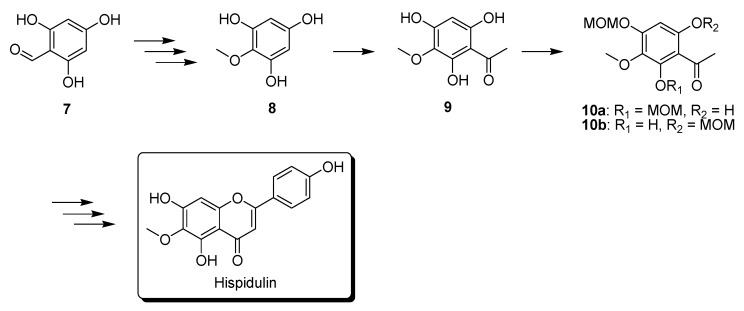
Synthesis of hispidulin in our previous study.

Kavvadias and coworkers developed a method synthesizing hispidulin from 2,4,6-trihydroxyacetophenone compound **6** in nine reaction steps (Scheme 2). However, the overall yield of this method is very limited (1.1%) [18]. We recently developed a feasible and reproducible approach for synthesizing hispidulin (Scheme 3) [21]. This method slightly improved the overall yield due to the low yield of Friedel–Crafts acetylation of compound **8** as well as unsatisfactory regioselective MOM protection of compound **9**. These facts motivated us to investigate an efficient and high-yield route to synthesize hispidulin.

Deuterium is a stable isotope of hydrogen. Because deuterium has a stronger chemical bond with carbon than hydrogen, deuterium-labelled compounds can affect the absorption, distribution, metabolism and toxicology of their counterpart compound [24,25]. The Food and Drug Administration (FDA) recently approved the first deuterated drug, deutetrabenazine, for treating involuntary writhing movements or chorea in Huntington’s disease [26]. Deuterium incorporation of tetrabenazine markedly increased its half-life and area under the curve (AUC) in plasma [27]. Study showed that the pig caecal microflora metabolized hispidulin to scutellarein due to *O*-demethylation at the C6 position [28]. This result suggested that replacement of OCH_3_ of this position of hispidulin using OCD_3_ may reduce its metabolic liability. Therefore, this study replaced C6-OCH_3_ in hispidulin with C6-OD_3_ to investigate whether such modification allows enhancement of the metabolic stability. Notably, introducing deuterium into hispidulin leads to a new chemical entity (NCE) that meets the criteria for a 505(b) (2) patent application [27].

The new hispidulin synthesis scheme developed in this study is more feasible compared to all methods previously reported. In particular, it had the highest overall yield, which may help to synthesize C6-OCH_3_-containing hispidulin derivatives. The same strategy can also be used to synthesize *d*-labelled hispidulin. The microsome stability of hispidulin and its deuterium counterpart were compared.

## 2. Results and Discussion

### 2.1. Retrosynthetic Analysis of Hispidulin

**Figure 2:**
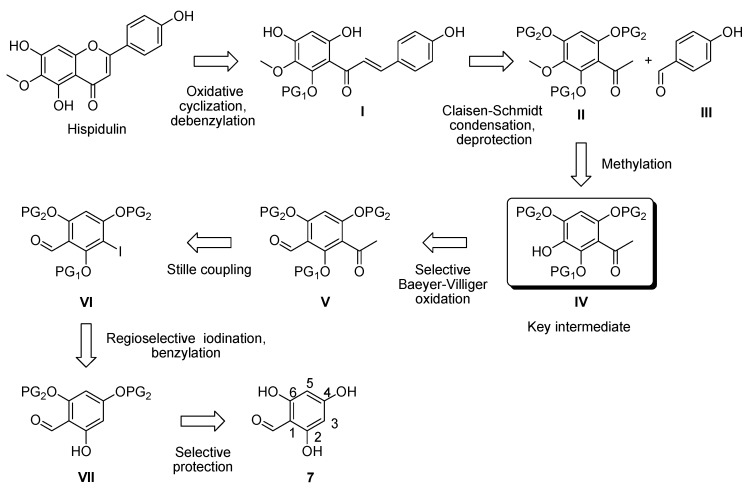
Retrosynthetic analysis of hispidulin.

Figure 2 shows the retrosynthetic analysis to successfully synthesize hispidulin. Hispidulin can be produced from compound **I** via debenzylation and oxidative cyclization. Compound **I** is derived from compound **II** and commercially available compound **III**, which are used to conduct Claisen–Schmidt condensation and deprotection of MOM (methoxymethoxy). Compound **II** in turn can be prepared from compound **IV** via methylation. Compound **IV** is considered to be the key intermediate that possesses two different protecting groups as well as acetyl and hydroxy moieties. Selective Bayer–Villiger reaction of compound **V** provided compound **IV**. Compound **V** is prepared from compound **VI** via Stille coupling. Benzylation and regioselective iodination of compound **VII** gives compound **VI**. Starting from commercially available compound **7**, Compound **VII** is synthesized via selective MOM protection.

### 2.2. Synthesis of Hispidulin

**Scheme 4:**
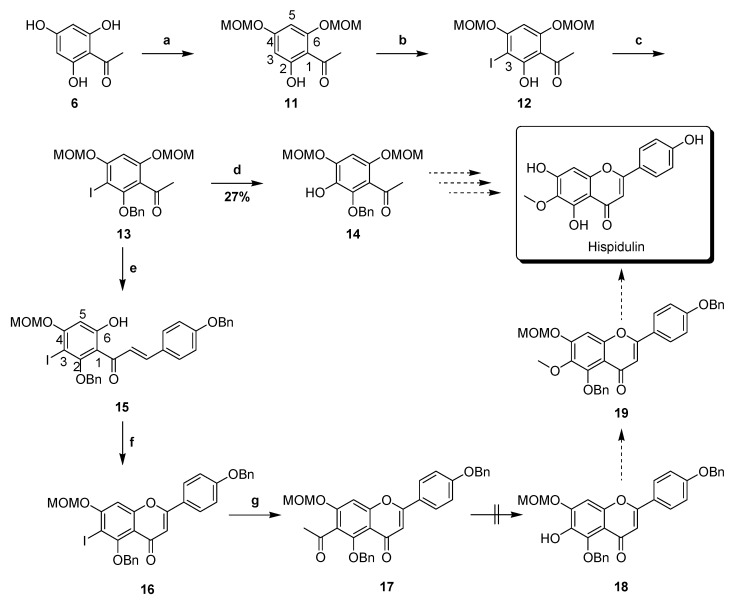
Initial attempt for the synthesis of hispidulin. *Reagents and conditions*: (**a**) MOMCl, DIPEA, CH_2_Cl_2_, 0 °C, 64%; (**b**) CF_3_COOAg, I_2_, CH_2_Cl_2_, 0 °C, 90%; (**c**) K_2_CO_3_, BnBr, DMF, 0 °C, 99%; (**d**) Pd_2_dba_3_, *t*BuXPhos, KOH, dioxane–H_2_O, 90 °C, 27%; (**e**) (1) BnOPhCHO, KOH, EtOH, H_2_O, 0 °C; (2) c-HCl, MeOH, THF, 0 °C, 77%; (**f**) I_2_, DMSO, 120 °C, 32%; (**g**) tributyl(1-ethoxyvinyl)tin, Pd(PPh_3_)_4_, dioxane, 100 °C, 61%.

Scheme 4 shows a synthetic approach originally developed for hispidulin. Using compound **6** as the starting material, selective MOM protection towards its C4- and C6-OH gave compound **11**. First, *N*-bromosuccinimide (NBS) was used for bromination of C3 of compound **11**; however, only undesirable C3 and C5 dibromination occurred. Alternatively, compound **11**, when reacted with I_2_ in the presence of Lewis acid CF_3_CO_2_Ag, provided selectively iodinated product **12** in the high yield (90%). The experimental result resulted from the difference of steric hindrance between C3 and C5 upon using the more bulky reagent I_2_. Benzylation of compound **12** generated compound **13**. Next, several reported oxidation methods were used [29,30,31] in attempts to convert the iodide group of compound **13** into phenol. These oxidation catalysts included CuI, Cu and Pd, as shown in Table 1. Reaction using Pd_2_dba_3_ coupled with the ligand 2-di-*tert*-butylphosphino-2′,4′,6′-triisopropylbiphenyl (*t*BuXPhos) gave compound **14**, but its yield was poor (27%). Despite the use of microwave irradiation, the yields were incapable of being improved. Instead, Claisen–Schmidt condensation of compound **13**, reacted with 4-(benzyloxy)benzaldehyde prior to selective deprotection of MOM group in the presence of HCl, gave chalcone **15**. The selective deprotection reaction can be manipulated by decreasing the reaction time to avoid further MOM deprotection of C4. Oxidative cyclization of compound **15** in the presence of catalytic I_2_ provided flavone **16**. Notably, excess I_2_ caused an undesirable retro-aldol reaction that gave C4- and C6-deprotected compound **13**. Treatment of catalytic Pd(PPh_3_)_4_ and tributyl(1-ethoxyvinyl)tin converted compound **16** to compound **17** through the Stille reaction. Baeyer–Villiger oxidation followed by basic hydrolysis of compound **17** by using H_2_O_2_ or *m*-chloroperoxybenzoic acid (MCPBA) [32] failed to give expected product **18**. 

**Scheme 5:**
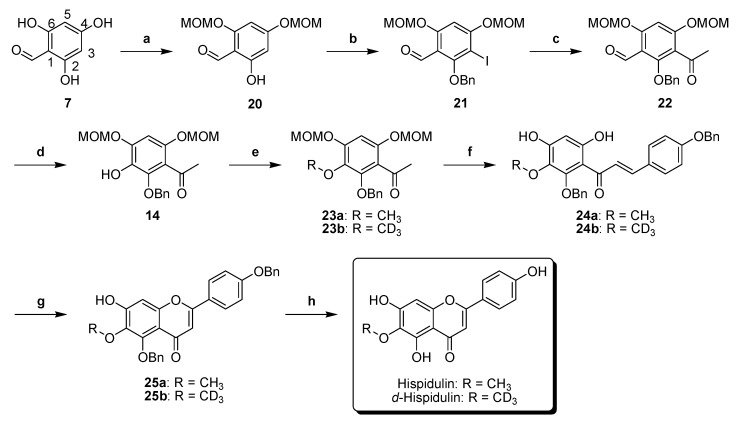
Synthesis of hispidulin and *d*-hispidulin. *Reagents and conditions*: (**a**) MOMCl, DIPEA, CH_2_Cl_2_, 0 °C, 80%; (**b**) (1) CF_3_COOAg, I_2_, CH_2_Cl_2_, 0 °C; (2) K_2_CO_3_, BnBr, DMF, 0 °C, 89%; (**c**) tributyl(1-ethoxyvinyl)tin, PdCl_2_(PPh_3_)_2_, toluene, 100 °C, 83%; (**d**) (1) MCPBA, CH_2_Cl_2_, 0 °C; (2) 10% NaOH_(aq)_, MeOH, 68%; (**e**) **23a**: CH_3_I, K_2_CO_3_, acetone, 56 °C, 92%; **23b**: CD_3_I, K_2_CO_3_, acetone, 56 °C, 93%; (**f**) (1) BnOPhCHO, KOH, EtOH, H_2_O, 0 °C; (2) c-HCl, MeOH, THF, 0 °C; **24a**: 92%, **24b**: 81%; (**g**) I_2_, DMSO, 120 °C, **25a**: 93%, **25b**: 79%; (**h**) 1M BCl_3_ in hexane; CH_2_Cl_2_, −80 °C, hispidulin: 85%, *d*-hispidulin: 80%.

**Figure 3:**
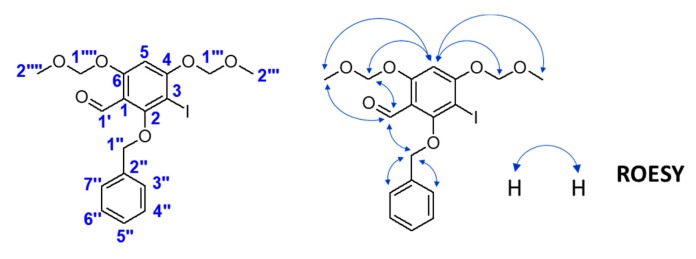
Key rotating frame nuclear Overhauser effect spectroscopy (ROESY) correlations of compound **21**.

**Figure 4:**
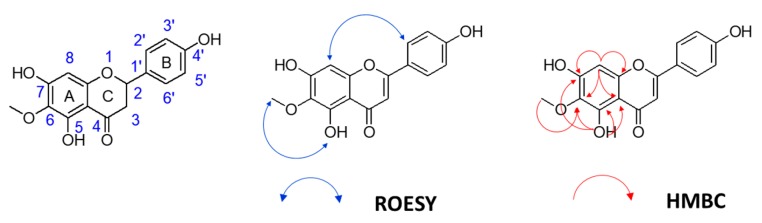
Key ROESY and HMBC correlations of hispidulin.

Thus, we developed a new synthesis (Scheme 5) for hispidulin using the retrosynthetic analysis as shown in Figure 2. First, 2,4,6-trihydroxybenzaldehyde **7** reacted with MOMCl gave the bis(methoxymethoxy)-protected compound **20**. Regioselective iodination of compound **20** prior to reaction with BnBr produced compound **21**. The chemical structure of compound **21** was confirmed by rotating frame nuclear Overhauser effect spectroscopy (ROESY) spectrum. Figure 3 shows the key correlation of H-5 (δ_H_ 6.84) to H-2′″ (δ_H_ 3.52), H-2″″ (δ_H_ 3.54), H-1′″ (δ_H_ 5.29) and H-1″″ (δ_H_ 5.32); and H-1′ (δ_H_ 10.30) to H-2″″ (δ_H_ 3.54), H-1″ (δ_H_ 4.99) and H-1″″ (δ_H_ 5.32) (Supplementary Materials). Stille coupling of compound **21** produced compound **22**. Table 2 shows how tributyl(1-ethoxyvinyl)tin was used to optimize Stille coupling. First, catalyst Pd(PPh_3_)_4_ was used in dioxane at 100 °C. The reaction had a satisfactory yield (68%), but the reaction time was up to 30 h. Replacement of the solvent by toluene led to the reaction time decreasing to 24 h. Further experiments using palladium catalysts such as PdCl_2_(PPh_3_)_2_ and Pd(dppf)Cl_2_ in dioxane or toluene showed that PdCl_2_(PPh_3_)_2_ significantly improved the yield and decreased the reaction time. In particular, PdCl_2_(PPh_3_)_2_ coupled with toluene not only gave the highest yield, but also had the lowest reaction time. Baeyer–Villiger oxidation and basic hydrolysis of compound **22** afforded compound **14**. Methylation of compound **14** using CH_3_I gave compound **23a**. Claisen–Schmidt condensation of compound **23a** with 4-(benzyloxy)benzaldehyde prior to MOM deprotection produced chalcone **24a**. Cyclization of compound **24a** in the presence of catalytic I_2_ generated flavone **25a**. Although debenzylation of compound **25a** catalyzed by 10% Pd–C did not yield hispidulin, a reaction using BCl_3_ at −80 °C successfully converted compound **25a** into hispidulin. The chemical structure of hispidulin was identified as judged by 2D-NMR analyses. Figure 4 shows the key correlation in the ROESY spectrum of hispidulin that 5-OH (δ_H_ 13.07) was correlated to 6-OMe′″ (δ_H_ 3.74) and H-8 (δ_H_ 6.59) was correlated to H-2′ and H-6′ (δ_H_ 7.92). Additionally, the HMBC spectrum showed that 5-OH (δ_H_ 13.07) correlated to C-5 (δ_C_ 152.8), C-6 (δ_C_ 131.4), C-7 (δ_C_ 157.3), C-9 (δ_C_ 152.4) and C-10 (δ_C_ 104.1); H-8 (δ_H_ 13.07) correlated to C-6 (δ_C_ 131.4), C-7 (δ_C_ 157.3), C-9 (δ_C_ 152.4) and C-10 (δ_C_ 104.1); 6-OMe-H (δ_H_ 3.74) correlated to C-6 (δ_C_ 131.4) (Supplementary Materials). The ^1^H- and ^13^C-NMR data of synthesized hispidulin were similar to those of hispidulin previously isolated (Supplementary Materials) [33].

### 2.3. Synthesis of d-Hispidulin

The synthesis of *d*-hispidulin is described in Scheme 5. Methylation of compound **14** using CD_3_I gave compound **23b**. The same method to hispidulin was used to synthesize *d*-labelled hispidulin starting from compound **23b**. Due to the absence of a proton signal of the CD_3_O group in the ^1^H-NMR spectra of the *d*-containing intermediate compounds **23b**, **24b**, **25b** and *d*-hispidulin, these compound structures were identified depending on the ^13^C-NMR spectra without ^1^H decoupling and the mass technique. The ^13^C-NMR spectra revealed a characteristic multiplet splitting pattern of the ^13^C signal for the CD_3_O group in compounds **23b**, **24b**, **25b** and *d*-hispidulin. The mass spectra also supported chemical structures of these *d*-labelled compounds. All synthesized compounds had an estimated purity of at least 98% as determined by HPLC analysis (Supplementary Materials).

### 2.4. Comparison of Hispidulin Synthesis Methods

Strategies for synthesizing hispidulin can be classified as semisynthesis and total synthesis strategies. The starting material used in most semisynthetic methods is scutellarin, which is a natural product. Table 3 shows that the semisynthesis routes had fewer reaction steps compared to the total synthesis methods; however, they need tedious isolation procedures for scutellarin, which limits the scale for further chemical modification. Furthermore, their overall yields are only 6.3–10.7% [20,22,23]. For total synthesis, Kavvadias and coworkers developed a nine-step synthesis approach. The starting material used in this method is commercially available 2,4,6-trihydroxyacetophenone. Although this method solves the issue of the source for starting material, its drawback is low overall yield [18]. We previously developed a feasible route of hispidulin synthesis that has an overall yield comparable to that of the method developed by Kavvadias and coworkers [21]. This present study further made the reaction steps more concise. In particular, the synthetic scheme showed the highest overall yield of all approaches to synthesize hispidulin.

### 2.5. Human Liver Microsome Stability

Metabolic stability is associated with susceptibility of compounds to biotransformation. Metabolic half-life (t_1/2_) and intrinsic clearance (CL_int_) was compared between hispidulin and *d*-hispidulin by testing these synthesized compounds in a human liver microsome stability assay. The study revealed that FDA-approved deuterated agent deutetrabenazine had a t_1/2_ (8.6 h) superior to tetrabenazine (4.8 h). In addition to t_1/2_, the AUC of deutetrabenazine (542 ng·hr/mg) was also higher than that of its counterpart compound (261 ng·hr/mg) [27]. In contrast, the experimental results indicated hispidulin and *d*-hispidulin had no significant difference in t_1/2_ and CL_int_ (Table 4), which suggested that the C6-OMe of hispidulin is resistant to be modified by the human liver microsome. The metabolic site of hispidulin in the human liver microsome is worthy of further study.

The experiments showed that this method of synthesizing hispidulin and its *d*-labelled derivative is highly feasible. Specifically, it increases overall yield compared to previous methods. The t_1/2_ and intrinsic clearance of these two compounds were identified as well. Overall, this synthetic route can be applied to produce 6-OMe-containing hispidulin derivatives as new chemical entities for investigating their biological activities.

## 3. Experimental Section

### 3.1. General Information

The NMR spectra (^1^H- and ^13^C-NMR, ROESY, HSQC and HMBC) were obtained with a Bruker AV500 using standard pulse programs. The MS data were recorded with a Finnigan Mat TSQ-7000 mass spectrometer (HR–ESI–MS) (Thermo, Ringoes, NJ, USA). The HPLC was performed on a C_18_ column (150 mm × 4.6 mm, Ascentis) by using an L-2130 pump (Hitachi, Ibaraki, Japan) and a UV/vis L-2420 detector (Hitachi, Ibaraki, Japan). The column chromatography was performed on silica gel (70-230 mesh, Merck, Darmstadt, Germany). All TLC analyses were performed on silica gel plates (KG60-F254, Merck, Darmstadt, Germany). Reagents and materials were used without further purification and chemicals were purchased from ACROS (Geel, Belgium). Dry dichloromethane was distilled from CaH_2_ under nitrogen atmosphere. MOMCl was acquired from TCI (Tokyo, Japan), and 2,4,6-trihydroxybenzaldehyde was purchased from Alfa Aesar (Heydham, UK).

### 3.2. Chemistry

*2-Hydroxy-4,6-bis(methoxymethoxy)benzaldehyde* (**20**). To a solution of compound **7** (10 g, 64.9 mmol) in CH_2_Cl_2_ (200 mL) was added DIPEA (28.3 mL, 162.2 mmol). The resulting mixture was stirred for 10 mins in an ice-bath under N_2_. MOMCl (10.8 mL, 142.7 mmol) was added dropwise to the reaction mixture by addition funnel. The reaction mixture was warmed to room temperature (rt) and stirred for 3 h. The mixture was concentrated in vacuo. The residue was diluted with EtOAc (100 mL) and washed with distilled H_2_O (3 × 80 mL). The organic layer was dried over Na_2_SO_4_, filtered and removed in vacuo. The residue was purified by silica gel chromatography (EtOAc:*n*-hexane = 1:9) to give compound **20** (12.3 g, 80%), a light-yellow microcrystalline powder; ^1^H-NMR (CDCl_3_, 300 MHz) δ 12.29 (1H, s), 10.16 (1H, s), 6.25 (1H, d, *J* = 2.1 Hz), 6.23 (1H, d, *J* = 2.1 Hz), 5.24 (2H, s), 5.18 (2H, s), 3.51 (3H, s), 3.47 (3H, s); ^13^C-NMR (CDCl_3_, 125 MHz) δ 192.1, 165.6, 165.5, 161.2, 106.9, 96.6, 94.6, 94.1, 94.0, 56.6, 56.5; HR–ESI–MS *m*/*z* 243.0858 [M + H]^+^ (calcd. for C_11_H_15_O_6_, 243.0863).

*2-Benzyloxy-3-iodo-4,6-bis(methoxymethoxy)benzaldehyde* (**21**). To a mixture of compound **20** (7.2 g, 29.6 mmol) and CF_3_CO_2_Ag (7.8 g, 35.5 mmol) in CH_2_Cl_2_ (150 mL) at 0 °C was added I_2_ (8.3 g, 32.5 mmol) in CH_2_Cl_2_ (200 mL) dropwise by an addition funnel over 2 h. The reaction mixture was warmed to rt and stirred for 3 h. Then, saturated Na_2_S_2_O_3(aq)_ (50 mL) was added and the mixture was washed with distilled H_2_O (3 × 100 mL). The organic layer was dried over Na_2_SO_4_, filtered and removed in vacuo. The residue was dissolved in DMF (100 mL) and then K_2_CO_3_ (7.2 g, 51.8 mmol) was added. Benzyl bromide (460 μL, 3.9 mmol) was added dropwise to the mixture by an addition funnel at 0 °C. The resulting solution was warmed to rt and stirred for 3 h. The mixture was diluted with EtOAc (300 mL) and washed with distilled H_2_O (3 × 00 mL). The organic layer was dried over Na_2_SO_4_, filtered and removed in vacuo. The residue was purified by silica gel chromatography (EtOAc:*n*-hexane = 1:5) to give compound **21** (12.1 g, 89%), a white microcrystalline powder; ^1^H-NMR (CDCl_3_, 300 MHz) δ 10.30 (1H, s), 7.66 (2H, dd, *J* = 1.8, 8.1 Hz), 7.45–7.36 (3H, m), 6.84 (1H, s), 5.32 (2H, s), 5.29 (2H, s), 4.99 (2H, s), 3.54 (3H, s), 3.52 (3H, s); ^13^C-NMR (CDCl_3_, 125 MHz) δ 187.2, 161.9, 161.8, 161.6, 136.1, 129.0, 128.5, 128.4, 115.5, 98.2, 95.2, 94.9, 78.5, 56.7; HR–ESI–MS *m*/*z* 459.0294 [M + H]^+^ (calcd. for C_18_H_20_O_6_I, 459.0299).

*3-Acetyl-2-benzyloxy-4,6-bis(methoxymethoxy)benzaldehyde* (**22**). To a mixture of compound **21** (5 g, 10.9 mmol) and PdCl_2_(PPh_3_)_2_ (766 mg, 1.1 mmol) in toluene (200 mL) was added tributyl(1-ethoxyvinyl)tin (5.5 mL, 16.4 mmol). The resulting solution was heated to 100 °C and stirred for 12 h. After cooling to rt, the reaction mixture was acidified with 1 M HCl (50 mL) and stirred for 30 min. The mixture was diluted with EtOAc (200 mL) and washed with distilled H_2_O (3 × 100 mL). The organic layer was dried over Na_2_SO_4_, filtered and concentrated in vacuo. The residue was purified by silica gel chromatography (EtOAc:*n*-hexane = 1:4) to give compound **22** (3.5 g, 83%), a yellow microcrystalline powder; ^1^H-NMR (CDCl_3_, 300 MHz) δ 10.37 (1H, s), 7.48 (2H, dd, *J* = 1.8, 7.8 Hz), 7.41–7.33 (3H, m), 6.80 (1H, s), 5.29 (2H, s), 5.24 (2H, s), 4.96 (2H, s), 3.53 (3H, s), 3.49 (3H, s), 2.43 (3H, s); ^13^C-NMR (CDCl_3_, 125 MHz) δ 200.9, 187.3, 162.1, 159.3, 158.4, 136.1, 129.0, 128.8, 128.6, 128.5, 128.4, 121.8, 114.3, 97.5, 95.0, 94.5, 79.1, 56.8, 32.5; HR–ESI–MS *m*/*z* 375.1432 [M + H]^+^ (calcd. for C_20_H_23_O_7_, 375.1438).

*2-Benzyloxy-3-hydroxy-4,6-bis(methoxymethoxy)acetophenone* (**14**). To a solution of 70% MCPBA (6.8 g, 27.6 mmol) in dry CH_2_Cl_2_ (100 mL) at 0 °C was added compound **22** (3.4g, 9.2 mmol) in CH_2_Cl_2_ (100 mL) dropwise by an addition funnel over 1 h. The resulting solution was warmed to rt and stirred for 8 h. Then, saturated Na_2_S_2_O_3(aq)_ (30 mL) was added and the reaction mixture was washed with distilled H_2_O (3 × 100 mL). The organic layer was dried over Na_2_SO_4_, filtered and concentrated in vacuo. The residue was dissolved in MeOH (80 mL) and 10% NaOH_(aq)_ (60 mL) was added. The resulting solution was stirred at rt for 1.5 h. The mixture was diluted with EtOAc (250 mL) and washed with distilled H_2_O (3 × 100 mL). The organic layer was dried over Na_2_SO_4_, filtered and concentrated in vacuo. The residue was purified by silica gel chromatography (EtOAc:*n*-hexane = 1:4) to give compound **14** (2.3 g, 68%), a light-yellow oil; ^1^H-NMR (CDCl_3_, 300 MHz) δ 7.44 (2H, dd, *J* = 1.8, 8.1 Hz), 7.40–7.32 (3H, m), 6.76 (1H, s), 5.61 (1H, s), 5.21 (2H, s), 5.08, (2H, s), 5.06 (2H, s), 3.53 (3H, s), 3.46 (3H, s), 2.45 (3H, s); ^13^C-NMR (CDCl_3_, 125 MHz) δ 201.3, 146.6, 146.2, 143.2, 136.9, 134.9, 128.5, 128.3, 122.1, 100.4, 96.0, 95.8, 76.3, 56.6, 56.3, 32.6; HR–ESI–MS *m*/*z* 363.1431 [M + H]^+^ (calcd. for C_19_H_23_O_7_, 363.1438).

*2-Benzyloxy-3-methoxy-4,6-bis(methoxymethoxy)acetophenone* (**23a**). To a mixture of compound **14** (587 mg, 1.6 mmol) and K_2_CO_3_ (1.1 g, 8.1 mmol) in acetone (20 mL) was added CH_3_I (0.5 mL, 8.1 mmol). The resulting solution was heated to 56 °C and stirred for 5 h. The reaction mixture was concentrated in vacuo, diluted with EtOAc (100 mL) and washed with distilled H_2_O (3 × 50 mL). The organic layer was dried over Na_2_SO_4_, filtered and concentrated in vacuo. The residue was purified by silica gel chromatography (EtOAc:*n*-hexane = 1:5) to give compound **23a** (558 mg, 92%), a white microcrystalline powder; ^1^H-NMR (CDCl_3_, 300 MHz) δ 7.42 (2H, dd, *J* = 2.0, 8.3 Hz), 7.40–7.32 (3H, m), 6.76 (1H, s), 5.23 (2H, s), 5.11 (2H, s), 5.07 (2H, s), 3.85 (3H, s), 3.53 (3H, s), 3.46 (3H, s), 2.38 (3H, s); ^13^C-NMR (CDCl_3_, 125 MHz) δ 200.6, 151.8, 149.4, 149.2, 137.9, 136.7, 128.1, 128.0, 127.7, 121.4, 99.6, 95.0, 94.9, 76.0, 60.8, 56.0, 55.9, 32.1; HR–ESI–MS *m*/*z* 377.1587 [M + H]^+^ (calcd. for C_20_H_25_O_7_, 377.1595).

*2-Benzyloxy-3-[^2^H_3_]-methoxy-4,6-bis(methoxymethoxy)acetophenone* (**23b**). Following the procedure as described for compound **23a**, reaction of compound **14** (1.8 g, 4.8 mmol), K_2_CO_3_ (3.3 g, 24.2 mmol), CD_3_I (1.5 mL, 24.2 mmol) in acetone (40 mL) gave compound **23b** (1.7 g, 93%), a white microcrystalline powder; ^1^H-NMR (CDCl_3_, 300 MHz) δ 7.42 (2H, dd, *J* = 2.1, 8.4 Hz), 7.39–7.31 (3H, m), 6.76 (1H, s), 5.23 (2H, s), 5.11 (2H, s), 5.07 (2H, s), 3.53 (3H, s), 3.46 (3H, s), 2.38 (3H, s); ^13^C-NMR (CDCl_3_, 125 MHz) δ 200.6, 151.8, 149.4, 149.2, 137.8, 136.7, 128.1, 128.0, 127.7, 121.4, 99.6, 95.0, 94.9, 76.0, 56.0, 55.9, 32.1; HR–ESI–MS *m*/*z* 380.1775 [M + H]^+^ (calcd. for C_20_H_22_D_3_O_7_, 380.1783).

*(E)-1-(2-Benzyloxy-4,6-dihydroxy-3-methoxyphenyl)-3-(4-benzyloxyphenyl)prop-2-en-1-one* (**24a**). To a solution of **23a** (545 mg, 1.45 mmol) and 4-benzyloxybenzaldyde (615 mg, 2.9 mmol) in EtOH (20 mL) was added KOH (813 mg, 14.5 mmol) in EtOH–H_2_O (3 mL:3 mL) dropwise by addition funnel at 0 °C over 30 min. The resulting solution was warmed to rt and stirred for 24 h. The mixture was diluted with distilled H_2_O (50 mL) and washed with EtOAc (3 × 50 mL). The organic layer was dried over Na_2_SO_4_, filtered and concentrated in vacuo. MeOH–THF (14.5 mL:14.5 mL) and 12 M HCl (0.7 mL) was added to the residue at 0 °C. The resulting solution was warmed to rt and stirred for 8 h. The reaction mixture was diluted with distilled H_2_O (50 mL) and washed with EtOAc (3 × 50 mL). The organic layer was dried over Na_2_SO_4_, filtered and concentrated in vacuo. The residue was purified by silica gel chromatography (EtOAc:*n*-hexane = 1:4) to give **24a** (641 mg, 92%), a microcrystalline powder; ^1^H-NMR (DMSO-*d*_6_, 300 MHz) δ 12.92 (1H, s), 10.52 (1H, s), 7.59 (2H, s), 7.48–7.45 (2H, m), 7.44–7.37 (5H, m), 7.32–7.29 (5H, m), 6.94 (2H, d, *J* = 8.8 Hz), 6.21 (1H, s), 5.16 (2H, s), 5.06 (2H, s), 3.77 (3H, s); ^13^C-NMR (DMSO-*d*_6_, 125 MHz) δ 192.0, 160.2, 159.6, 157.7, 153.1, 143.0, 136.7, 136.6, 134.4, 130.3, 128.5, 128.4, 128.3, 128.2, 128.0, 127.8, 127.4, 124.6, 115.2, 109.0, 99.7, 75.7, 69.4, 60.7; HR–ESI–MS *m*/*z* 483.1795 [M + H]^+^ (calcd. for C_30_H_27_O_6_, 483.1802).

*(E)-1-(2-Benzyloxy-4,6-dihydroxy-3-[^2^H_3_]methoxyphenyl)-3-(4-benzyloxyphenyl)prop-2-en-1-one* (**24b**). According to the procedure as described for compound **24a**, reaction of compound **23b** (1.0 g, 2.6 mmol), 4-benzyloxybenzaldyde (1.1 g, 5.3 mmol) and KOH (1.5 g, 26.4 mmol) in EtOH (60 mL) followed by treatment of 12 M HCl (1.3 mL) and MeOH–THF (26.4 mL:26.4 mL) gave compound **24b** (1.0 g, 81%), a yellow microcrystalline powder; ^1^H-NMR (DMSO-*d*_6_, 300 MHz) δ 12.95 (1H, s), 10.54 (1H, s), 7.59 (2H, s), 7.48–7.45 (2H, m), 7.44–7.36 (5H, m), 7.33–7.29 (5H, m), 6.94 (2H, d, *J* = 8.8 Hz), 6.22 (1H, s), 5.16 (2H, s), 5.06 (2H, s); ^13^C-NMR (DMSO-*d*_6_, 125 MHz) δ 192.0, 160.2, 159.7, 157.7, 153.2, 143.0, 136.7, 136.6, 134.4, 130.3, 128.5, 128.4, 128.3, 128.2, 128.0, 127.8, 127.4, 124.6, 115.2, 109.0, 99.7, 75.7, 69.4; HR–ESI–MS *m*/*z* 486.1983 [M + H]^+^ (calcd. for C_30_H_24_D_3_O_6_, 486.1990).

*4′-Benzyloxy-6-methoxy-5-benzyloxy-7-hydroxyflavone* (**25a**). To a solution of compound **24a** (598 mg, 1.2 mmol) in dry DMSO (100 mL) was added I_2_ (32 mg, 0.1 mmol) in DMSO (3 mL) dropwise by syringe. The resulting solution was heated to 120 °C, and stirred for 2 h. After cooling to rt, saturated Na_2_S_2_O_3(aq)_ (10 mL) was added to the reaction mixture. The mixture was diluted with EtOAc (100 mL) and wash with distilled H_2_O (3 × 50 mL). The organic layer was dried over Na_2_SO_4_, filtered and concentrated in vacuo. The residue was purified by silica gel chromatography (EtOAc:*n*-hexane = 1:3) to give **25a** (555 mg, 93%), a yellow microcrystalline powder; ^1^H-NMR (DMSO-*d*_6_, 300 MHz) δ 10.76 (1H, s), 7.98 (2H, d, *J* = 8.9 Hz), 7.61 (2H, d, *J* = 7.0 Hz), 7.48 (2H, d, *J* = 7.0 Hz), 7.44–7.34 (6H, m), 7.18 (2H, d, *J* = 8.9 Hz), 6.91 (1H, s), 6.67 (1H, s), 5.22 (2H, s), 5.00 (2H, s), 3.75 (3H, s); ^13^C-NMR (DMSO-*d*_6_, 125 MHz) δ 175.8, 160.9, 160.1, 156.1, 153.8, 150.7, 139.6, 137.6, 136.6, 128.5, 128.3, 128.1, 128.0, 127.8, 123.4, 115.3, 111.4, 106.0, 100.1, 75.6, 69.5, 60.9; HR–ESI–MS *m*/*z* 481.1637 [M + H]^+^ (calcd. for C_30_H_25_O_6_, 481.1646).

*4′-Benzyloxy-6-[^2^H_3_]methoxy-5-benzyloxy-7-hydroxyflavone* (**25b**). Following the procedure as described for compound **25a**, reaction of compound **24b** (849 mg, 1.7 mmol) with I_2_ (44 mg, 0.2 mmol) in DMSO (120 mL) gave compound **25b** (664 mg, 79%), a yellow microcrystalline powder; ^1^H-NMR (DMSO-*d*_6_, 300 MHz) δ 10.75 (1H, s), 7.98 (2H, d, *J* = 8.9 Hz), 7.61 (2H, d, *J* = 6.8 Hz), 7.48 (2H, dd, *J* = 1.8, 8.4 Hz), 7.44–7.34 (6H, m), 7.18 (2H, d, *J* = 8.9 Hz), 6.91 (1H, s), 6.67 (1H, s), 5.22 (2H, s), 5.01 (2H, s); ^13^C-NMR (DMSO-*d*_6_, 125 MHz) δ 175.8, 160.9, 160.1, 156.1, 153.7, 150.7, 139.5, 137.6, 136.6, 128.5, 128.3, 128.1, 128.0, 127.8, 123.4, 115.3, 111.4, 106.0, 100.1, 75.6, 69.5; HR–ESI–MS *m*/*z* 484.1827 [M + H]^+^ (calcd. for C_30_H_22_D_3_O_6_, 484.1834).

*Hispidulin.* To a solution of compound **25a** (301 mg, 0.6 mmol) in dry CH_2_Cl_2_ (40 mL) was added 1 M BCl_3_ (2.5 mL, 2.5 mmol) in dry CH_2_Cl_2_ (5 mL) dropwise by syringe at −78 °C over 20 min. The resulting solution was stirred for 1 h. The reaction mixture was diluted with distilled H_2_O (50 mL) and washed with EtOAc (3 × 50 mL). The organic layer was dried over Na_2_SO_4_, filtered and concentrated in vacuo. The residue was purified by silica gel chromatography (EtOAc:*n*-hexane = 1:2) to give hispidulin (160 mg, 85%), a yellow microcrystalline powder; ^1^H-NMR (DMSO-*d*_6_, 500 MHz) δ 13.07 (1H, s, 5-OH), 10.73 (1H, s, 7-OH), 10.38 (1H, s, 4′-OH), 7.92 (2H, d, *J* = 8.9 Hz, H-2′, H-6′), 6.92 (2H, d, *J* = 8.9 Hz, H-3′, H-5′), 6.77 (1H, s, H-3), 6.59 (1H, s, H-8), 3.74 (3H, s, 6-OMe); ^13^C-NMR (DMSO-*d*_6_, 125 MHz) δ 182.2 (C-4), 163.9 (C-2), 161.2 (C-4′), 157.3 (C-7), 152.8 (C-5), 152.4 (C-9), 131.4 (C-6), 128.5 (C-2’, C-6′), 121.2 (C-1), 116.0 (C-3′, C-5′), 104.1 (C-10), 102.4 (C-3), 94.3 (C-8), 60.0 (6-OMe); HR–ESI–MS *m*/*z* 301.0702 [M + H]^+^ (calcd. for C_16_H_13_O_6_, 301.0707).

*d-Hispidulin.* Following the procedure as described for hispidulin, reaction of compound **25b** (536 mg, 1.1 mmol) in CH_2_Cl_2_ (75 mL) with 1 M BCl_3_ (4.4 mL, 4.4 mmol) in CH_2_Cl_2_ (8.8 mL) gave *d*-hispidulin (268 mg, 80%), a yellow microcrystalline powder; ^1^H-NMR (DMSO-*d*_6_, 500 MHz) δ 13.07 (1H, s, 5-OH), 10.70 (1H, s, 7-OH), 10.36 (1H, s, 4′-OH), 7.91 (2H, d, *J* = 8.9 Hz, H-2′, H-6′), 6.92 (2H, d, *J* = 8.9 Hz, H-3′, H-5′), 6.76 (1H, s, H-3), 6.58 (1H, s, H-8); ^13^C-NMR (DMSO-*d*_6_, 125 MHz) δ 182.1 (C-4), 163.8 (C-2), 161.2 (C-4’), 157.2 (C-7), 152.8 (C-5), 152.4 (C-9), 131.3 (C-6), 128.5 (C-2′, C-6′), 121.2 (C-1), 116.0 (C-3′, C-5′), 104.1 (C-10), 102.4 (C-3), 94.2 (C-8); HR–ESI–MS *m*/*z* 304.0888 [M + H]^+^ (calcd. for C_16_H_10_D_3_O_6_, 304.0895).

### 3.3. Human Liver Microsome Stability Assay

Mixed-gender human liver microsomes (Lot # 1210347) were purchased from XenoTech. The reaction mixture minus NADPH was prepared as described below. The test compounds were added into the reaction mixture at a final concentration of 1 μM. A separate reaction with the control compound, testosterone, was run simultaneously with the reactions with the test compounds. An aliquot of the reaction mixture (without cofactor) was equilibrated in a shaking water bath at 37 °C for 3 min. After addition of cofactor to initiate the reaction, the mixture was incubated in a shaking water bath at 37 °C. Aliquots (100 μL) were withdrawn at 0, 10, 20, 30 and 60 min for the test compounds and testosterone. The reaction was terminated by immediately combining the tested compounds and testosterone samples with 400 μL of ice-cold 50/50 acetonitrile (ACN)/H_2_O containing 0.1% formic acid and internal standard. The samples were then mixed and centrifuged to precipitate proteins. All samples were assayed by LC-MS/MS using electrospray ionization. The peak area response ratio (PARR) to internal standard was compared to the PARR at time 0 to determine the percent remaining at each time point. The values for half-life (t_1/2_) and intrinsic clearance (CL_int_) of the tested compounds were determined by Absorption System Corp. Half-life calculated using GraphPad software was fitted to a single-phase exponential decay equation.

## Figures and Tables

**Table 1:** Attempts towards the oxidation of aryl halide **13**.

Entry	Catalyst	Ligand	Base	Solvent	T (°C)	Yield (%)
1	CuI	1,10-Phenanthroline	KOH	DMSO–H_2_O	100	trace
2	Cu	-	NaOH	H_2_O	100	trace
3	Cu	-	NaOH	DMSO–H_2_O	100	trace
4	Pd_2_dba_3_	*t*BuXPhos	KOH	Dioxane–H_2_O	100	27
5	Pd_2_dba_3_	*t*BuXPhos	KOH	Dioxane–H_2_O	70	14
6	Pd_2_dba_3_	*t*BuXPhos	KOH	Dioxane–H_2_O	100 ^1^	26
7	Pd_2_dba_3_	*t*BuXPhos	KOH	Dioxane–H_2_O	100 ^2^	23
8	Pd_2_dba_3_	*t*BuXPhos	KOH	DMF	100 ^1^	trace

^1^ Microwave reaction: 100 W, 20 min; ^2^ microwave reaction: 120 W, 20 min.

**Table 2:** Optimization of reaction condition for Stille coupling of compound **21**.

Entry	Catalyst	Solvent	Yield (%)	Reaction Time (h)
1	Pd(PPh_3_)_4_	Dioxane	68	30
2	Pd(PPh_3_)_4_	Toluene	70	24
3	PdCl_2_(PPh_3_)_2_	Dioxane	73	20
4	PdCl_2_(PPh_3_)_2_	Toluene	83	10
5	Pd(dppf)Cl_2_	Dioxane	34	78
6	Pd(dppf)Cl_2_	Toluene	53	43

**Table 3:** Comparison of hispidulin synthesis methods.

Research Group	Synthesis Route	Reaction Steps	Overall Yield (%)
Shen and coworkers	Semisynthesis	Seven	10.7
Lin and coworkers	Semisynthesis	Seven	7.1
Zhang and coworkers	Semisynthesis	Four	6.3
Kavvadias and coworkers	Total synthesis	Nine	1.1
Chao and coworkers	Total synthesis	Ten	1.6
This study	Total synthesis	Eight	26.9

**Table 4:** Human liver microsome stability of hispidulin and *d*-hispidulin.

Compound	Half-Life (min)	CL_int_^1^ (mL/min/mg Protein)
Hispidulin	46	0.0298
*d*-Hispidulin	43	0.0325
Testosterone	19	0.0727

^1^ Intrinsic clearance (CL_int_) was calculated based on CL_int_ = k/P, where k is the elimination rate constant and P is the protein concentration in the incubation.

## Supplementary Materials

The following are available online. ^1^H- and ^13^C-NMR spectra and HPLC chromatogram of all compounds synthesized; ^1^H- and ^13^C-NMR spectra comparison of experimental and reported hispidulin; ROESY spectra of compound **21**; and ROESY, HMQC and HMBC spectra of final products hispidulin and *d*-hispidulin.
