# Supplementary material for: Total Synthesis and Metabolic Stability of Hispidulin and Its d-Labelled Derivative
Source: Molecules. 2017 Nov 4;22(11):1897. doi: 10.3390/molecules22111897 (PMC6150239; doi:10.3390/molecules22111897)
Supplement: Supplementary file 1 [file molecules-22-01897-s001.pdf]

Supporting Information

Total Synthesis and Metabolic Stability of Hispidulin and its *d*-Labelled Derivative

Liang-Chieh Chen, Kai-Cheng Hsu, Lih-Chu Chiou, Hui-Ju Tseng, and Wei-Jan Huang*

Table of Contents

Table S1. Comparison of ^{1}H and ^{13}C NMR data of synthesis and natural hispidulin. 4

Figure S1. ^{1}H NMR ($CDCl_3$, 300 MHz) spectrum of compound **20** 5

Figure S2. ^{13}C NMR ($CDCl_3$, 125 MHz) spectrum of compound **20** 5

Figure S3. ^{1}H NMR ($CDCl_3$, 300 MHz) spectrum of compound **21** 6

Figure S4. ^{13}C NMR ($CDCl_3$, 125 MHz) spectrum of compound **21** 6

Figure S5. ROESY spectrum of compound **21** 7

Figure S6. ^{1}H NMR ($CDCl_3$, 300 MHz) spectrum of compound **22** 7

Figure S7. ^{13}C NMR ($CDCl_3$, 125 MHz) spectrum of compound **22** 8

Figure S8. ^{1}H NMR ($CDCl_3$, 300 MHz) spectrum of compound **14** 8

Figure S9. ^{13}C NMR ($CDCl_3$, 125 MHz) spectrum of compound **14** 9

Figure S10. ^{1}H NMR ($CDCl_3$, 300 MHz) spectrum of compound **23a** 9

Figure S11. ^{13}C NMR ($CDCl_3$, 125 MHz) spectrum of compound **23a** 10

Figure S12. ^{1}H NMR ($CDCl_3$, 300 MHz) spectrum of compound **23b** 10

Figure S13. ^{13}C NMR ($CDCl_3$, 125 MHz) spectrum of compound **23b** 11

Figure S14. ^{1}H NMR ($CDCl_3$, 300 MHz) spectrum of compound **24a** 11

Figure S15. ^{13}C NMR ($CDCl_3$, 125 MHz) spectrum of compound **24a** 12

Figure S16. ^{1}H NMR (DMSO-d_6, 300 MHz) spectrum of compound **24b** 12

Figure S17. ^{13}C NMR (DMSO-d_6, 125 MHz) spectrum of compound **24b** 13

Figure S18. ^{1}H NMR ($CDCl_3$, 300 MHz) spectrum of compound **25a** 13

Figure S19. ^{13}C NMR (DMSO-d_6, 125 MHz) spectrum of compound **25a** 14

Figure S20. ^{1}H NMR (DMSO-d_6, 300 MHz) spectrum of compound **25b** 14

Figure S21. ^{13}C NMR (DMSO-d_6, 125 MHz) spectrum of compound **25b** 15

Figure S22. ^{1}H NMR (DMSO-d_6, 500 MHz) spectrum of hispidulin 15

Figure S23. ^{13}C NMR (DMSO-d_6, 125 MHz) spectrum of hispidulin 16

Figure S24. ROESY spectrum of hispidulin 16

Figure S25. HSQC spectrum of hispidulin 17

Figure S26. HMBC spectrum of hispidulin ... 17

Figure S27. HMBC spectrum of hispidulin ... 18

Figure S28. HMBC spectrum of hispidulin ... 18

Figure S29. ^{1}H NMR (DMSO-d_6, 500 MHz) spectrum of *d*-hispidulin ... 19

Figure S30. ^{13}C NMR (DMSO-d_6, 125 MHz) spectrum of *d*-hispidulin ... 19

Figure S31. ROESY spectrum of *d*-hispidulin ... 20

Figure S32. HSQC spectrum of *d*-hispidulin ... 20

Figure S33. HSQC spectrum of *d*-hispidulin ... 21

Figure S34. HMBC spectrum of *d*-hispidulin ... 21

Figure S35. HMBC spectrum of *d*-hispidulin ... 22

Figure S36. HMBC spectrum of *d*-hispidulin ... 22

Figure S37. HPLC chromatogram of compound **20** ... 23

Figure S38. HPLC chromatogram of compound **21** ... 23

Figure S39. HPLC chromatogram of compound **22** ... 23

Figure S40. HPLC chromatogram of compound **14** ... 24

Figure S41. HPLC chromatogram of compound **23a** ... 24

Figure S42. HPLC chromatogram of compound **23b** ... 24

Figure S43. HPLC chromatogram of compound **24a** ... 25

Figure S44. HPLC chromatogram of compound **24b** ... 25

Figure S45. HPLC chromatogram of compound **25a** ... 25

Figure S46. HPLC chromatogram of compound **25b** ... 26

Figure S47. HPLC chromatogram of hispidulin ... 26

Figure S48. HPLC chromatogram of *d*-hispidulin ... 26

Table S1. Comparison of ^{1}H and ^{13}C NMR data of hispidulin synthesized in this study and in literature.

Position	This study [1]		Literature [2]	
	δ_H, m (J/Hz)	δ_C	δ_H, m (J/Hz)	δ_C
**2**		163.8, qC		163.8, qC
**3**	6.77, s	102.4, CH	6.75, s	102.4, CH
**4**		182.2, qC		182.1, qC
**5**		152.8, qC		152.8, qC
**5-OH**	13.07, s		13.05	
**6**		131.4, qC		131.4, qC
**6-OMe**	3.74, s	60.0	3.73, s	59.9
**7**		157.3, qC		157.3, qC
**7-OH**	10.73, s			
**8**	6.59, s	94.3, CH	6.57, s	94.2, CH
**9**		152.4, qC		152.4, qC
**10**		104.1, qC		104.0, qC
**1′**		121.2, qC		121.2, qC
**2′, 6′**	7.92, d (8.9)	128.5, CH	7.90, d (8.8)	128.4, CH
**3′, 5′**	6.92, d (8.9)	116.0, CH	6.90, d (8.8)	115.9, CH
**4′**		161.2, qC		161.2, qC
**4′-OH**	10.38, s			

[1] ^{1}H NMR (500 MHz, DMSO-d_6); ^{13}C NMR (125 MHz, DMSO-d_6); [2] ^{1}H NMR (400 MHz, DMSO-d_6); ^{13}C NMR (100 MHz, DMSO-d_6); data obtained from reference 33.

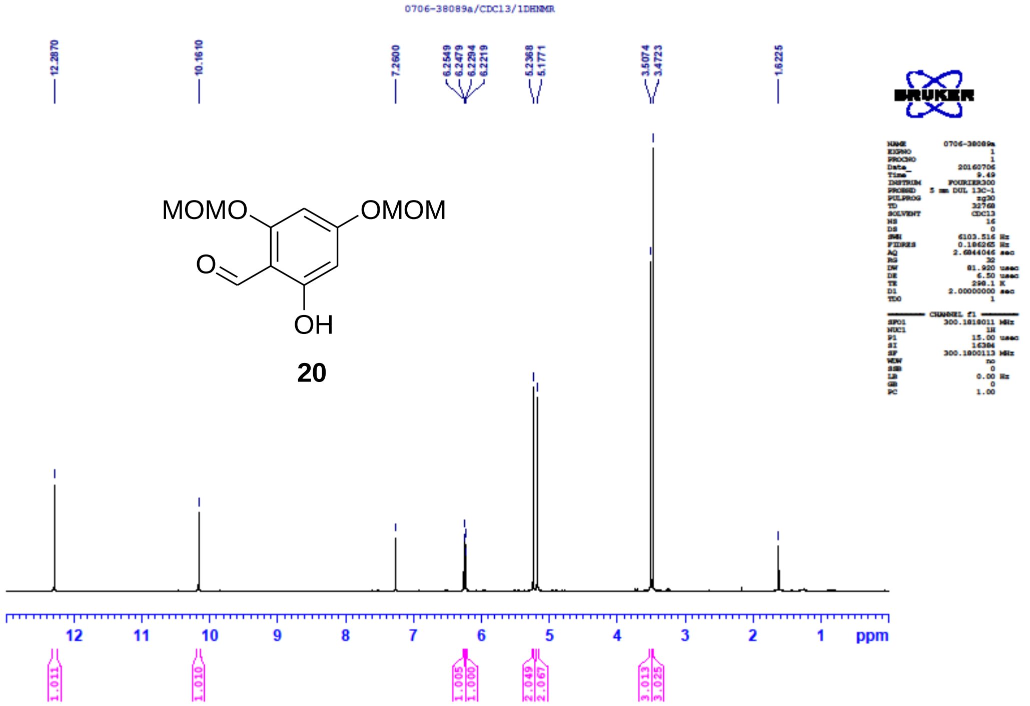

Figure S1. ^{1}H NMR ($CDCl_3$, 300 MHz) spectrum of compound **20**

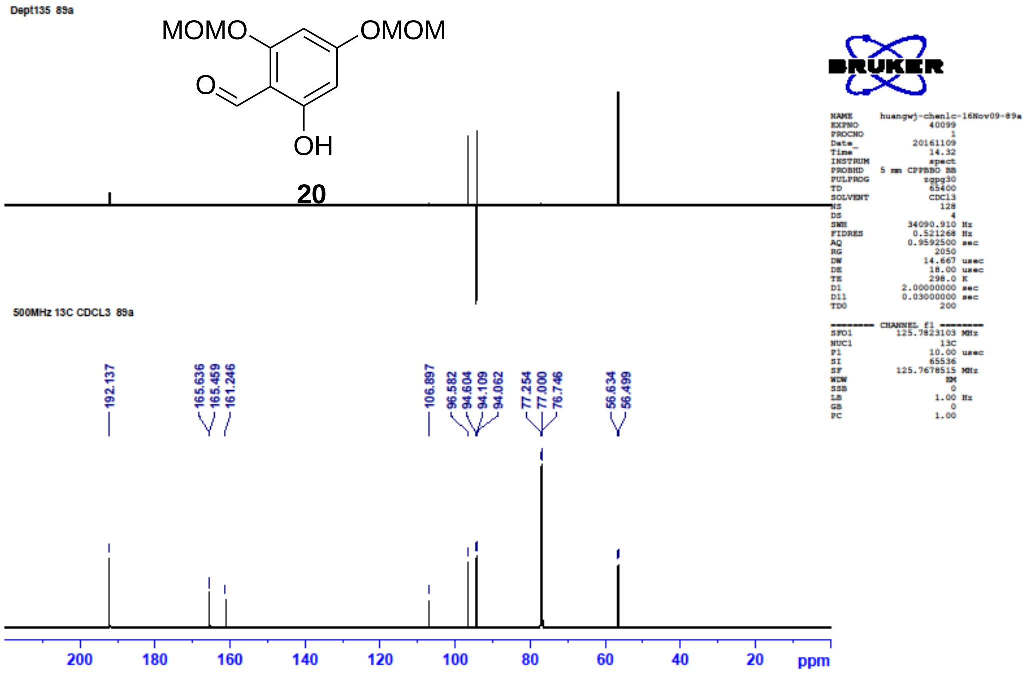

Figure S2. ^{13}C NMR ($CDCl_3$, 125 MHz) spectrum of compound **20**

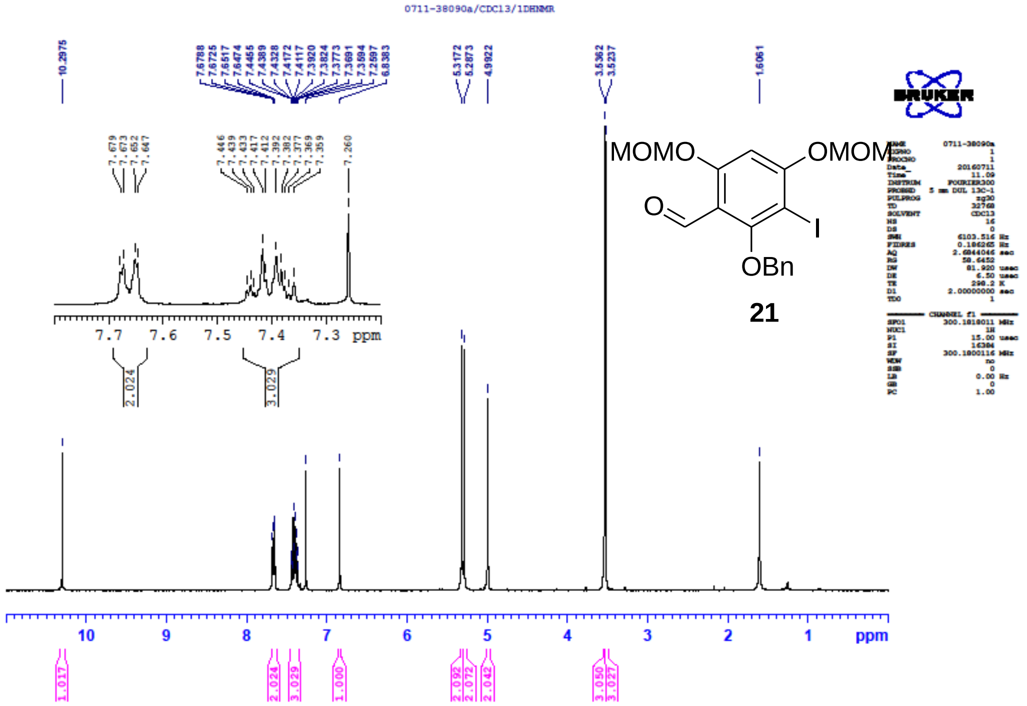

Figure S3. ^{1}H NMR ($CDCl_3$, 300 MHz) spectrum of compound **21**

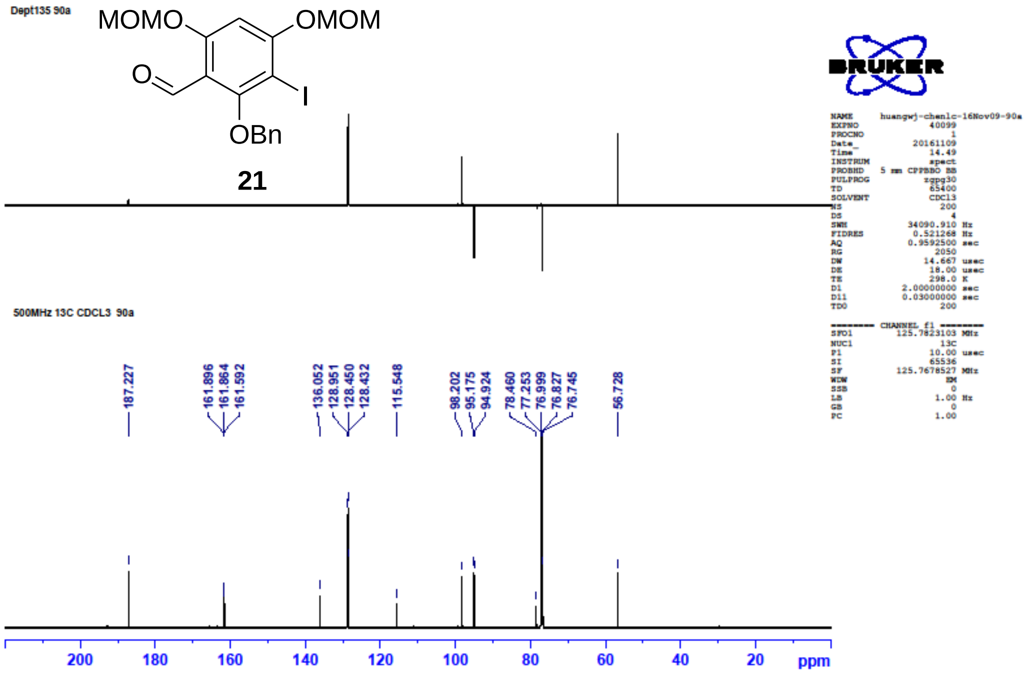

Figure S4. ^{13}C NMR ($CDCl_3$, 125 MHz) spectrum of compound **21**

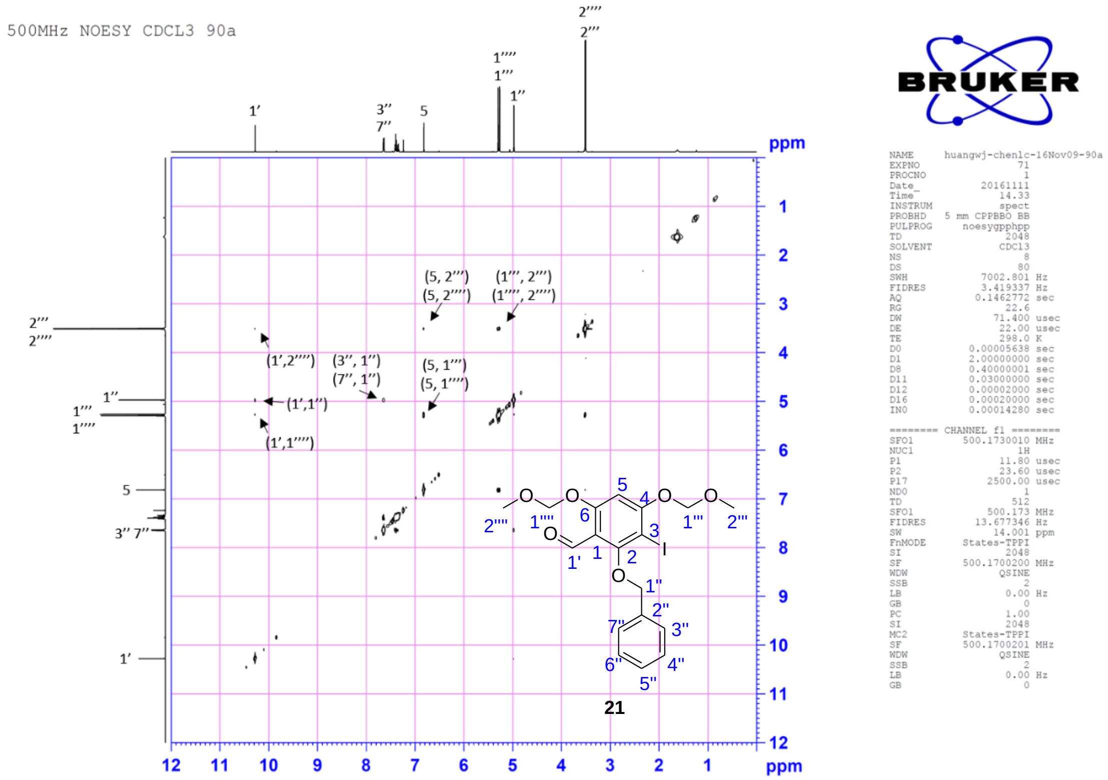

Figure S5. ROESY spectrum of compound **21**

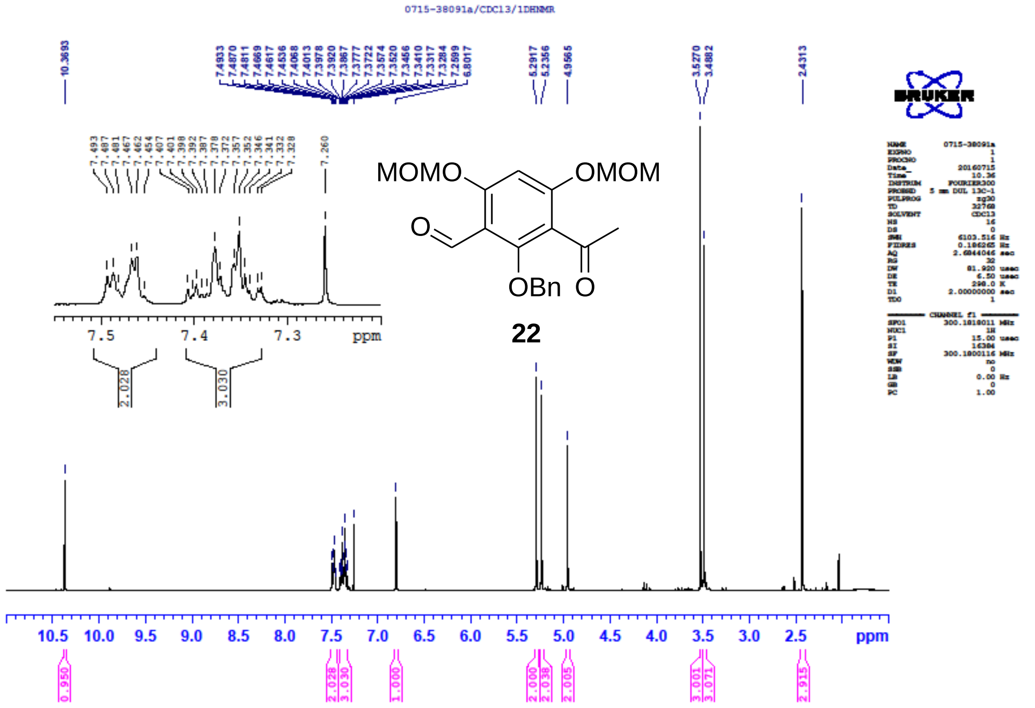

Figure S6. ^1H NMR ($CDCl_3$, 300 MHz) spectrum of compound **22**

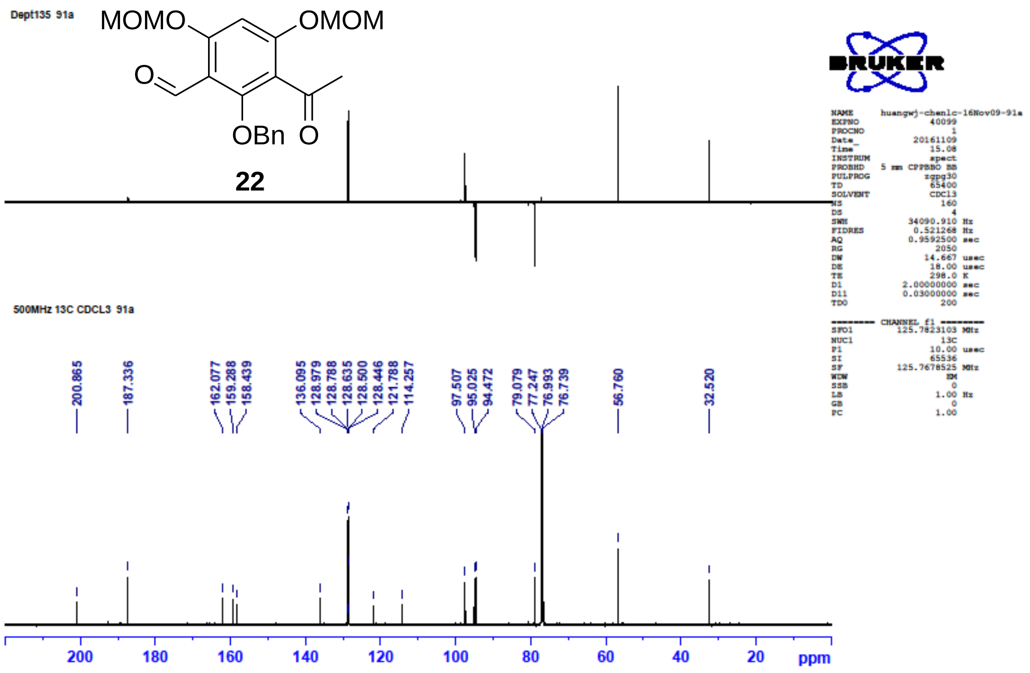

Figure S7. ^{13}C NMR ($CDCl_3$, 125 MHz) spectrum of compound **22**

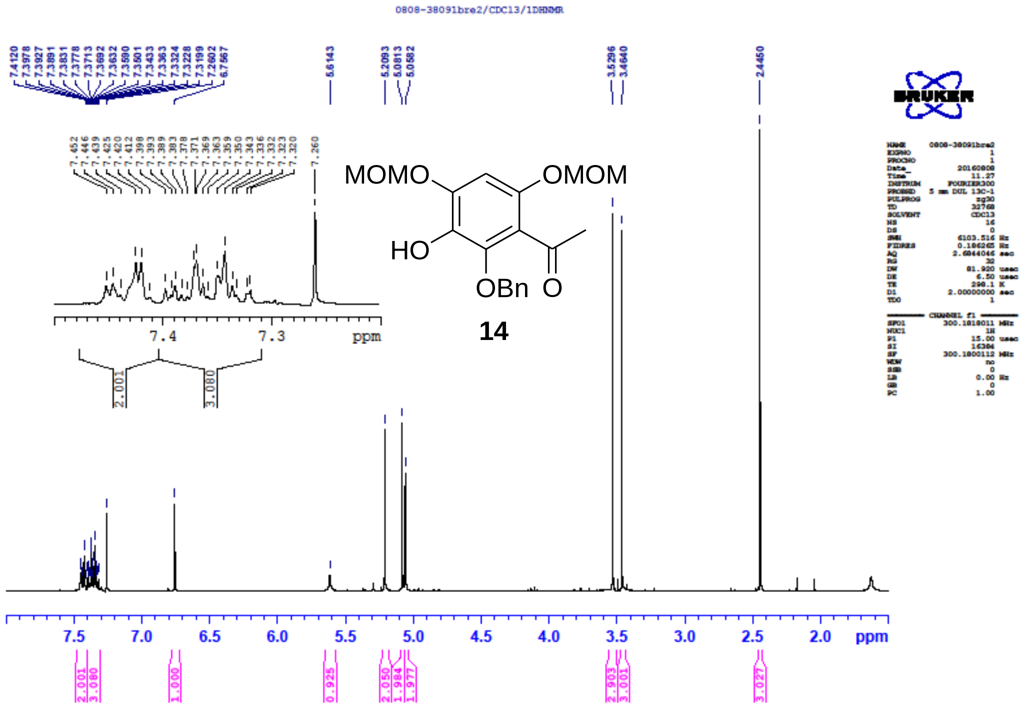

Figure S8. ^1H NMR ($CDCl_3$, 300 MHz) spectrum of compound **14**

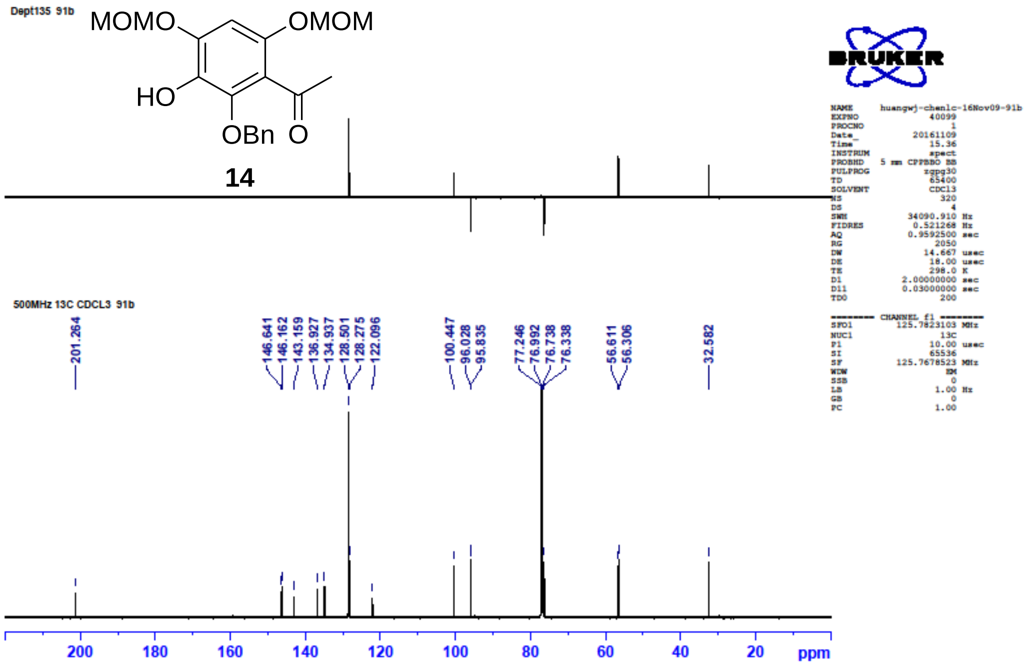

Figure S9. ^{13}C NMR ($CDCl_3$, 125 MHz) spectrum of compound **14**

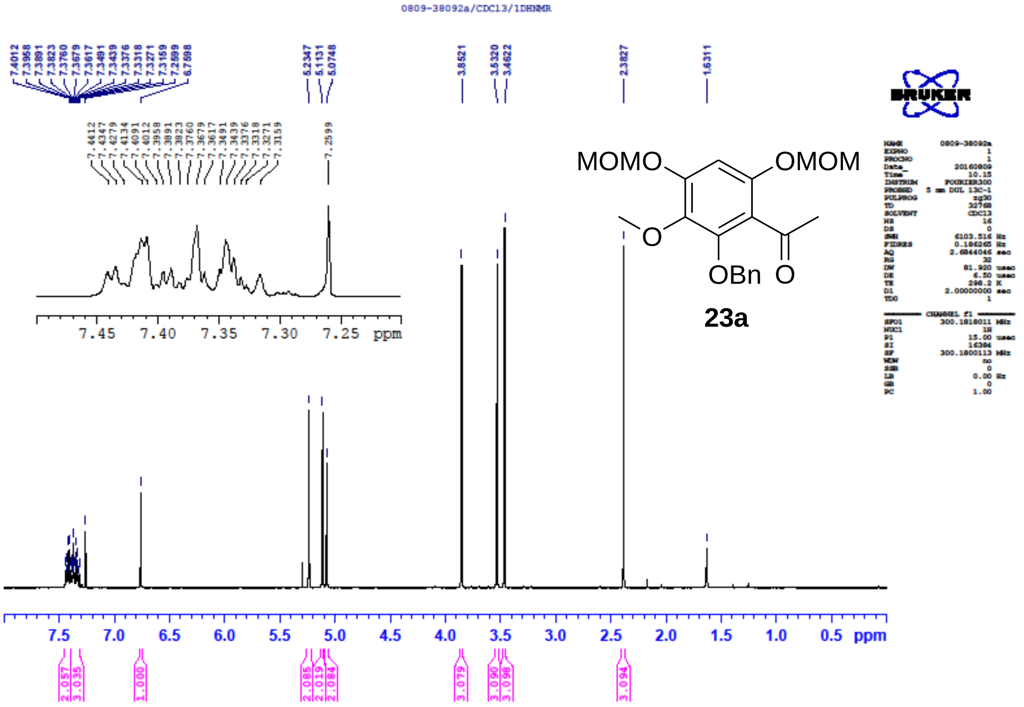

Figure S10. ^{1}H NMR ($CDCl_3$, 300 MHz) spectrum of compound **23a**

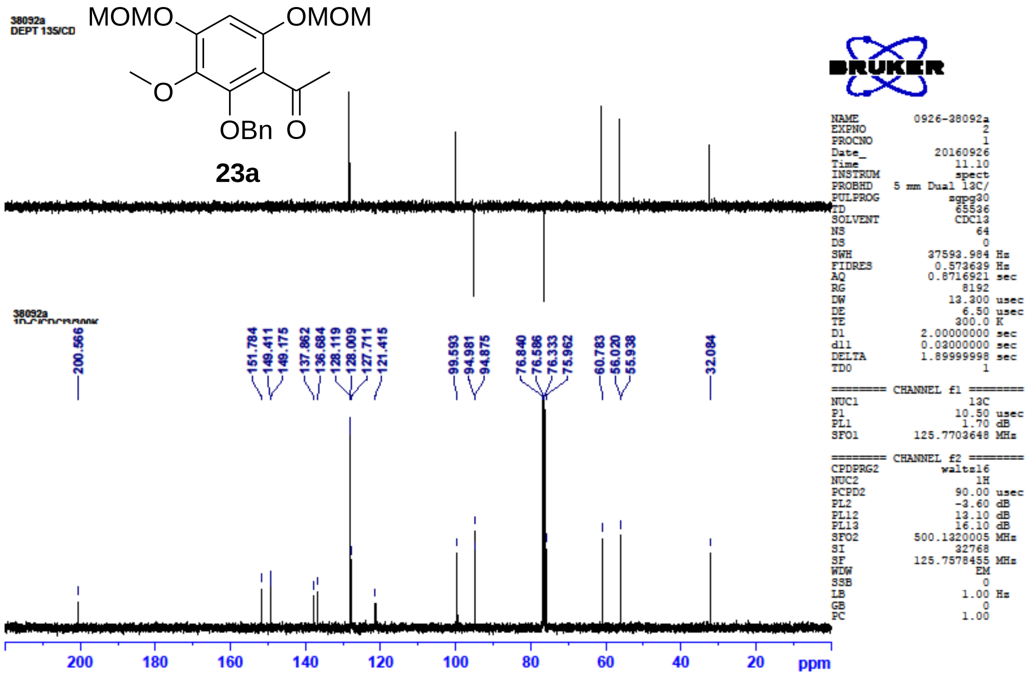

Figure S11. ^{13}C NMR ($CDCl_3$, 125 MHz) spectrum of compound **23a**

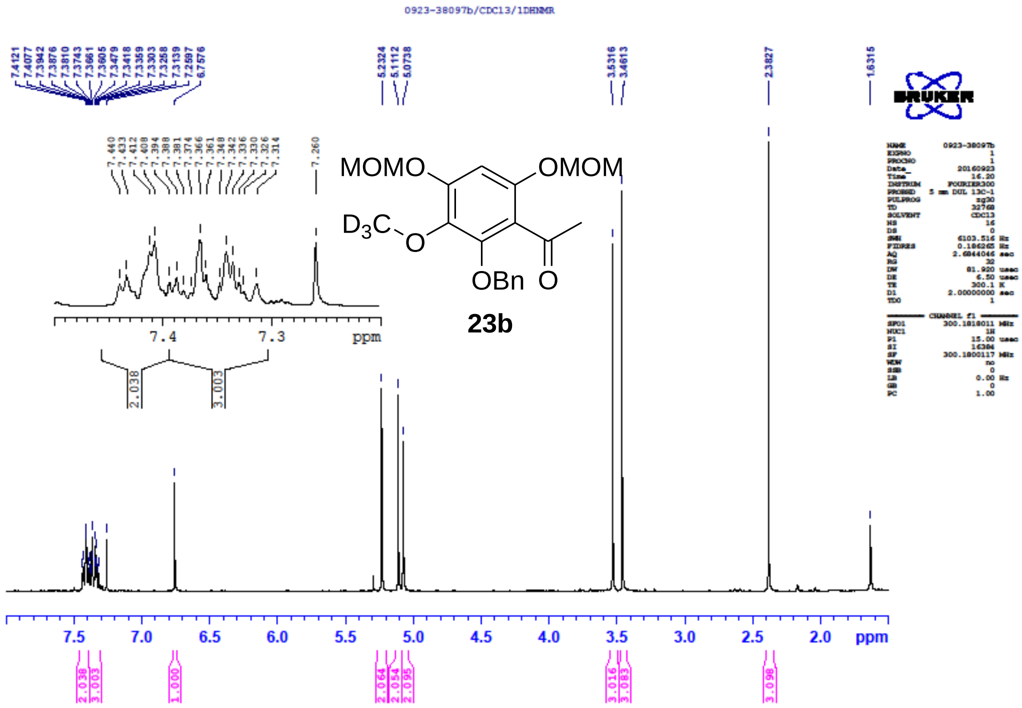

Figure S12. ^{1}H NMR ($CDCl_3$, 300 MHz) spectrum of compound **23b**

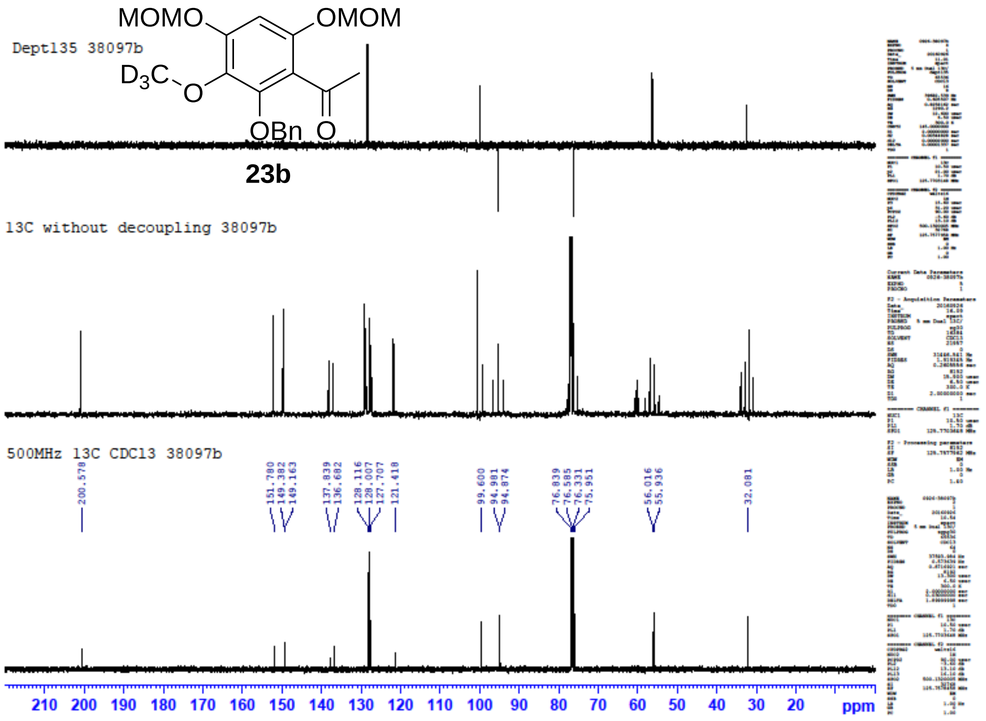

Figure S13. ^{13}C NMR ($CDCl_3$, 125 MHz) spectrum of compound **23b**

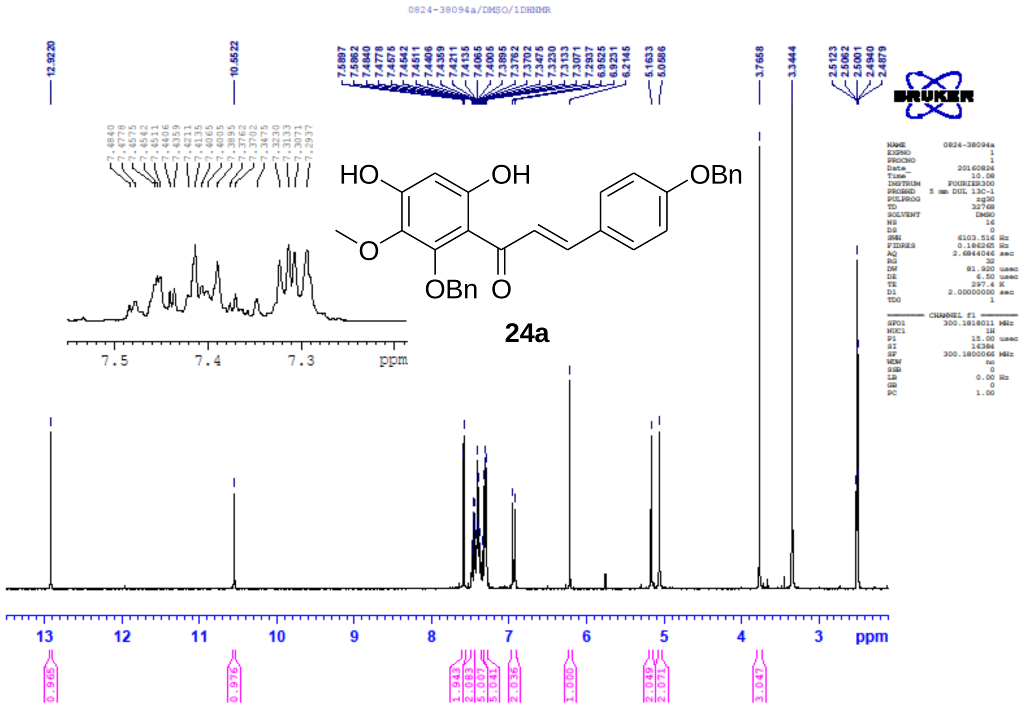

Figure S14. ^1H NMR ($CDCl_3$, 300 MHz) spectrum of compound **24a**

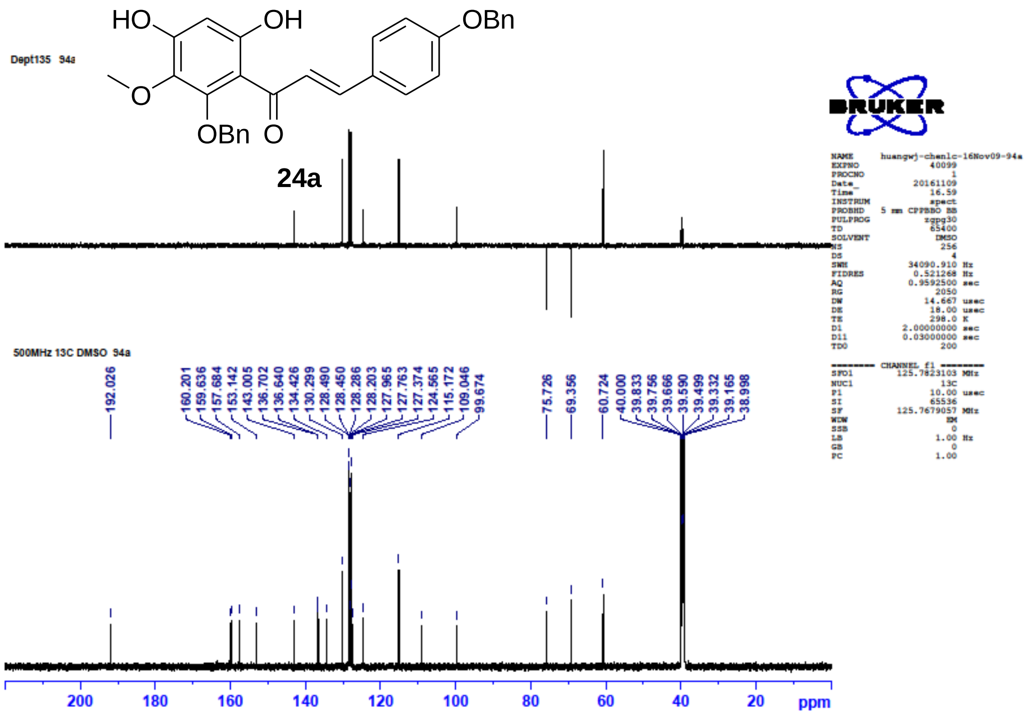

Figure S15. ^{13}C NMR ($CDCl_3$, 125 MHz) spectrum of compound **24a**

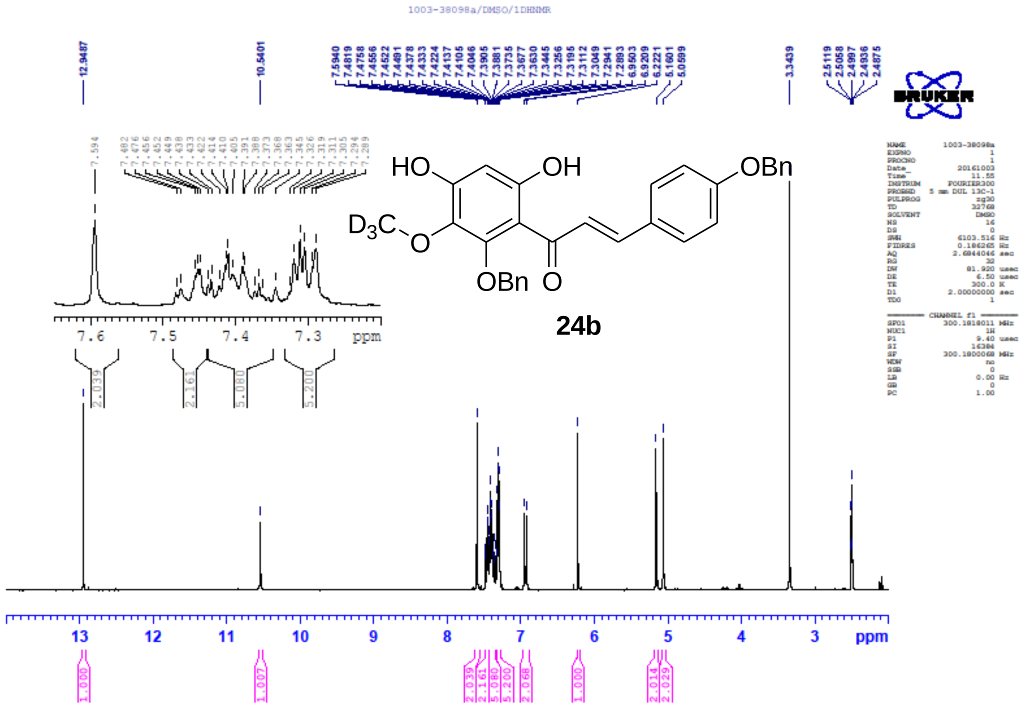

Figure S16. ^{1}H NMR (DMSO-*d*$_6$, 300 MHz) spectrum of compound **24b**

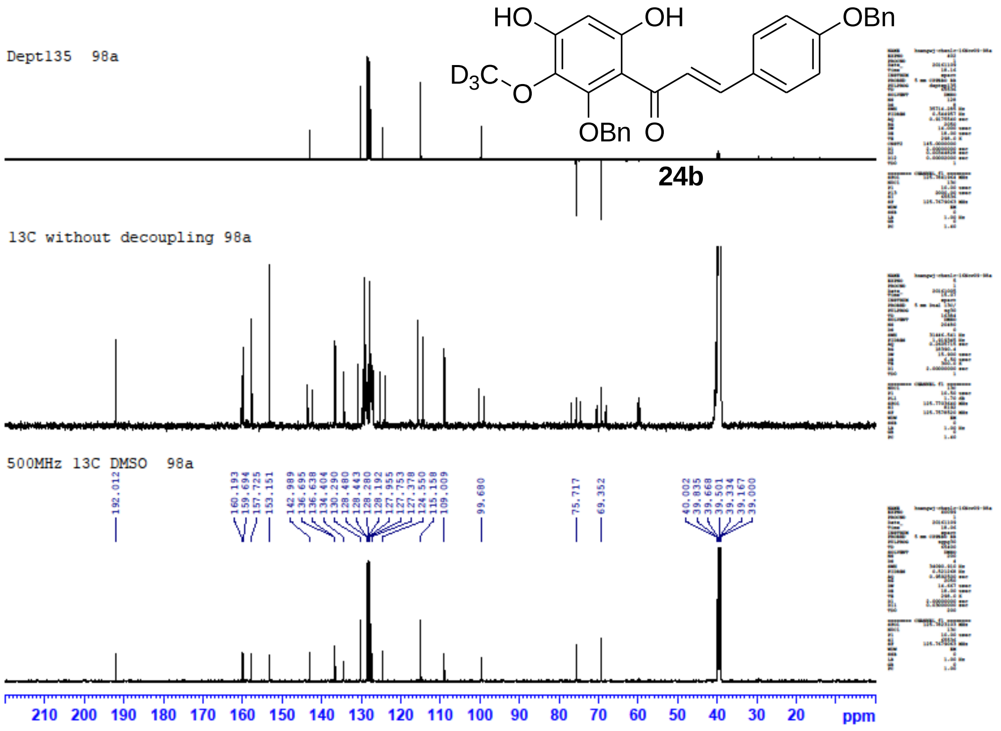

Figure S17. ^{13}C NMR (DMSO-d_6, 125 MHz) spectrum of compound **24b**

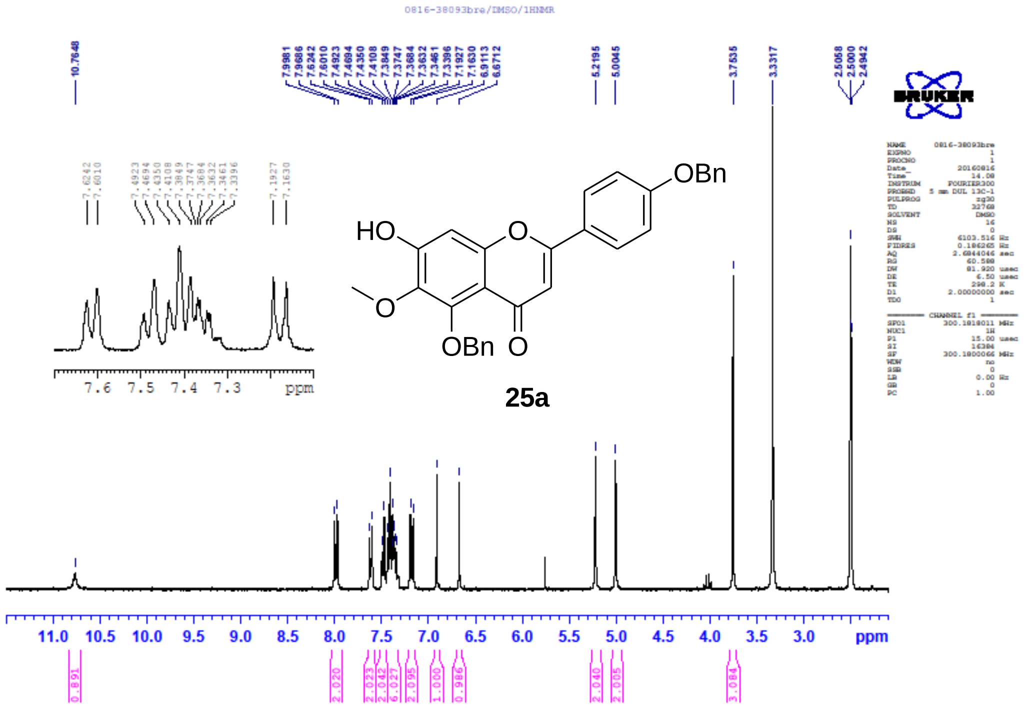

Figure S18. ^{1}H NMR ($CDCl_3$, 300 MHz) spectrum of compound **25a**

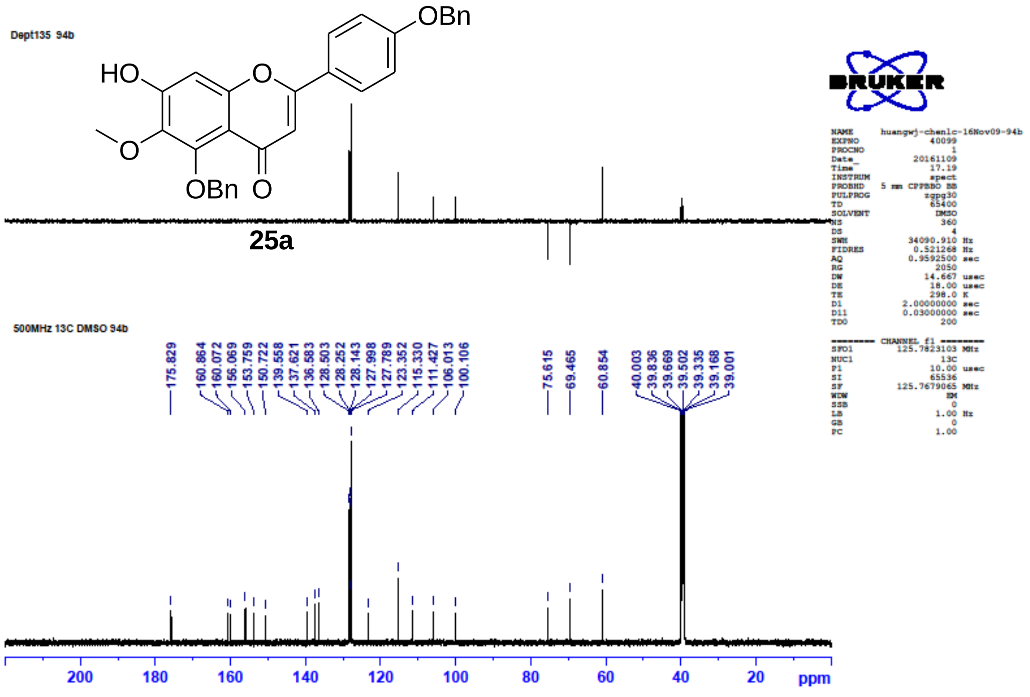

Figure S19. ^{13}C NMR (DMSO-d_6, 125 MHz) spectrum of compound **25a**

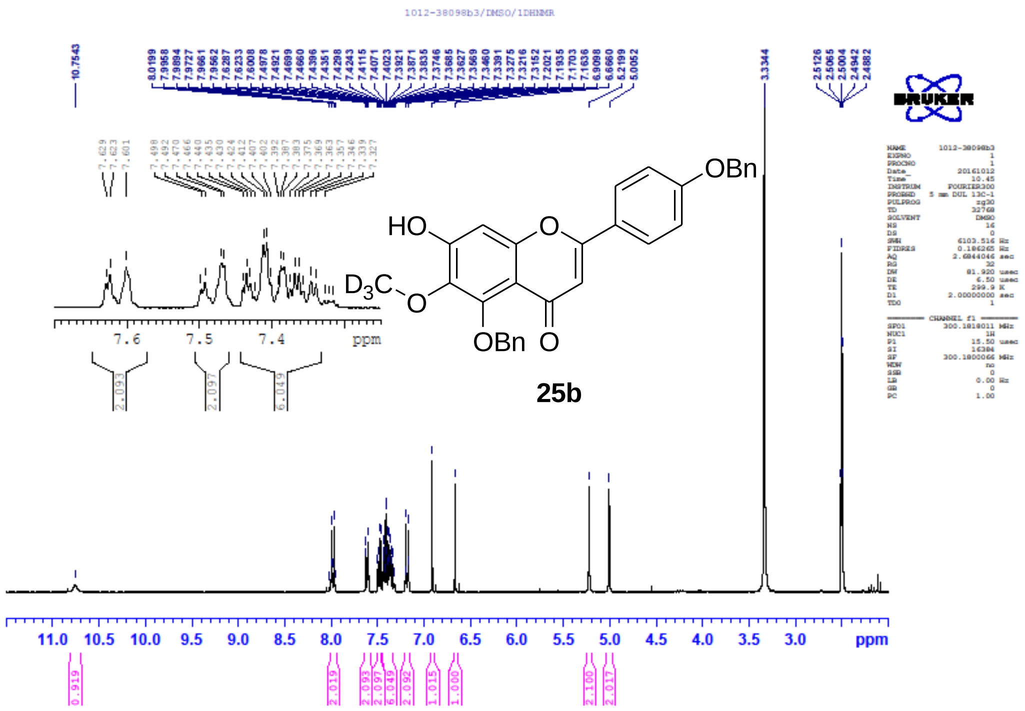

Figure S20. ^{1}H NMR (DMSO-d_6, 300 MHz) spectrum of compound **25b**

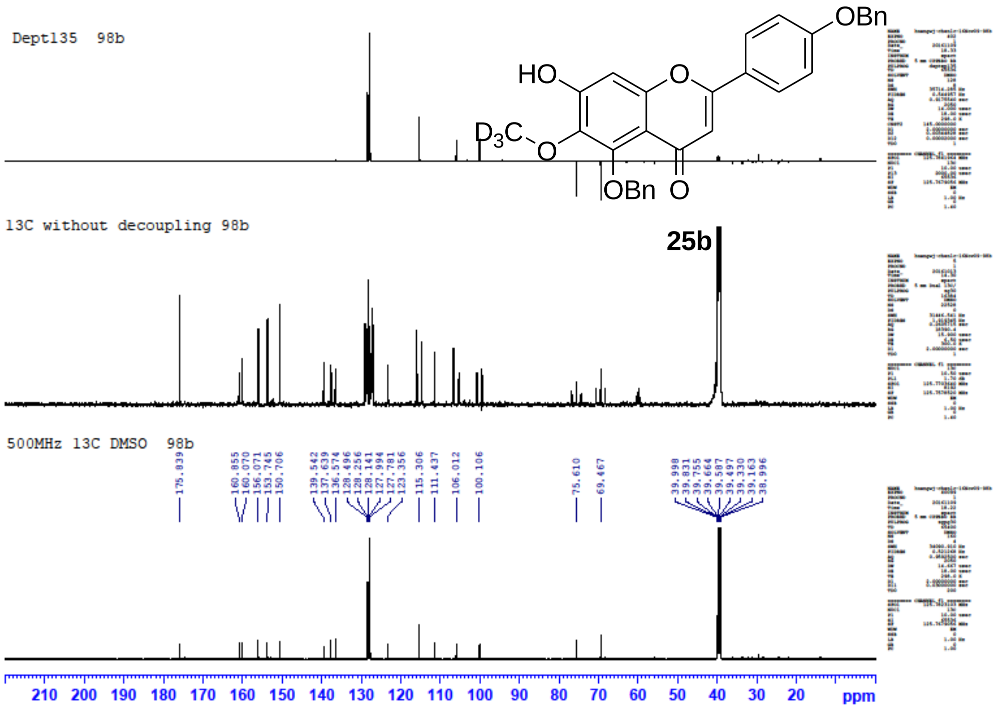

Figure S21. ^{13}C NMR (DMSO-d_6, 125 MHz) spectrum of compound **25b**

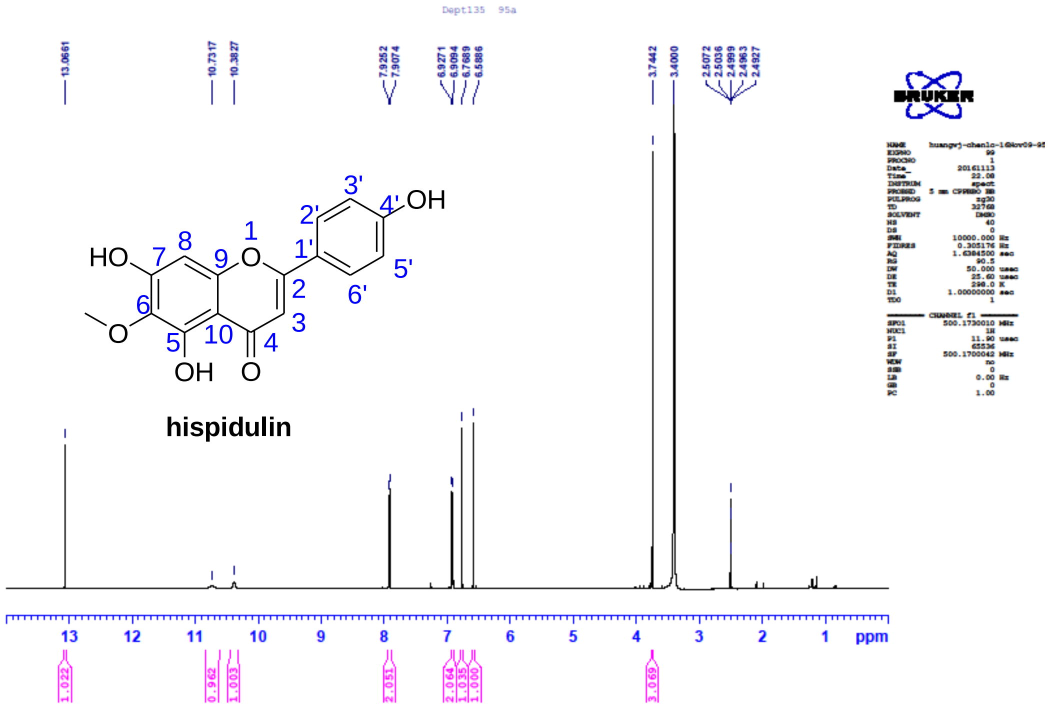

Figure S22. ^{1}H NMR (DMSO-d_6, 500 MHz) spectrum of hispidulin

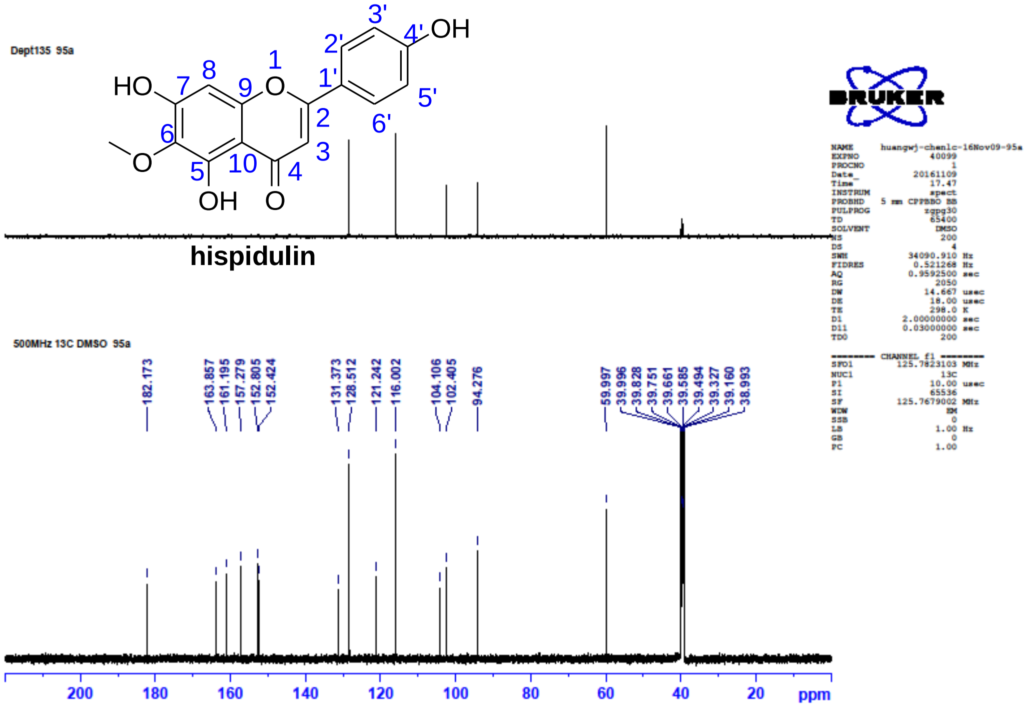

Figure S23. ^{13}C NMR (DMSO-d_6, 125 MHz) spectrum of hispidulin

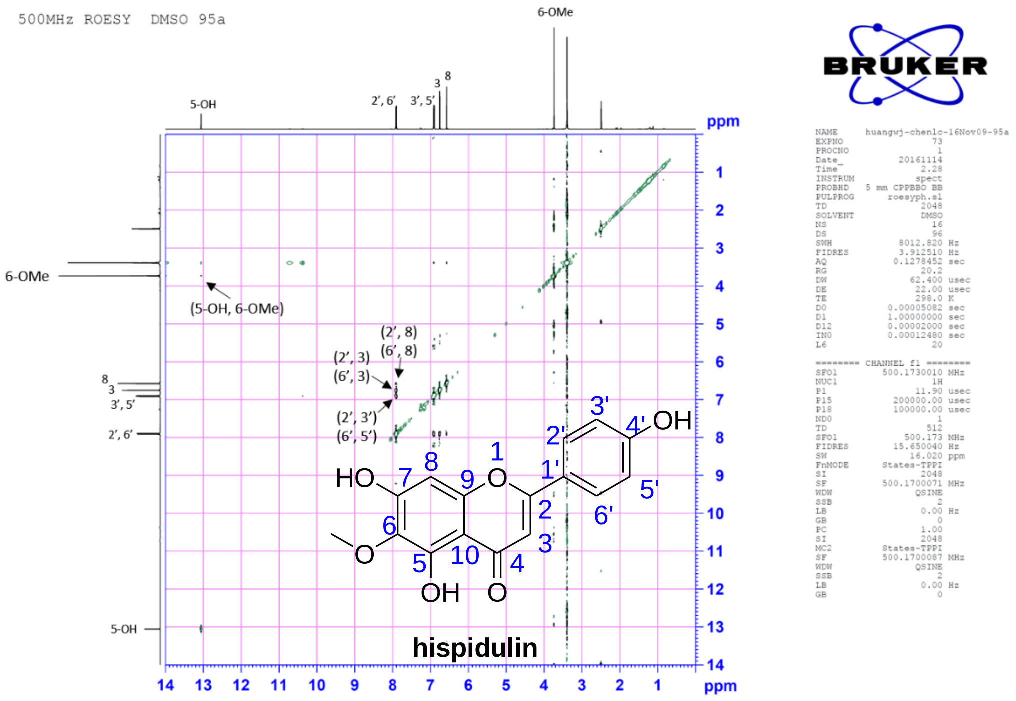

Figure S24. ROESY spectrum of hispidulin

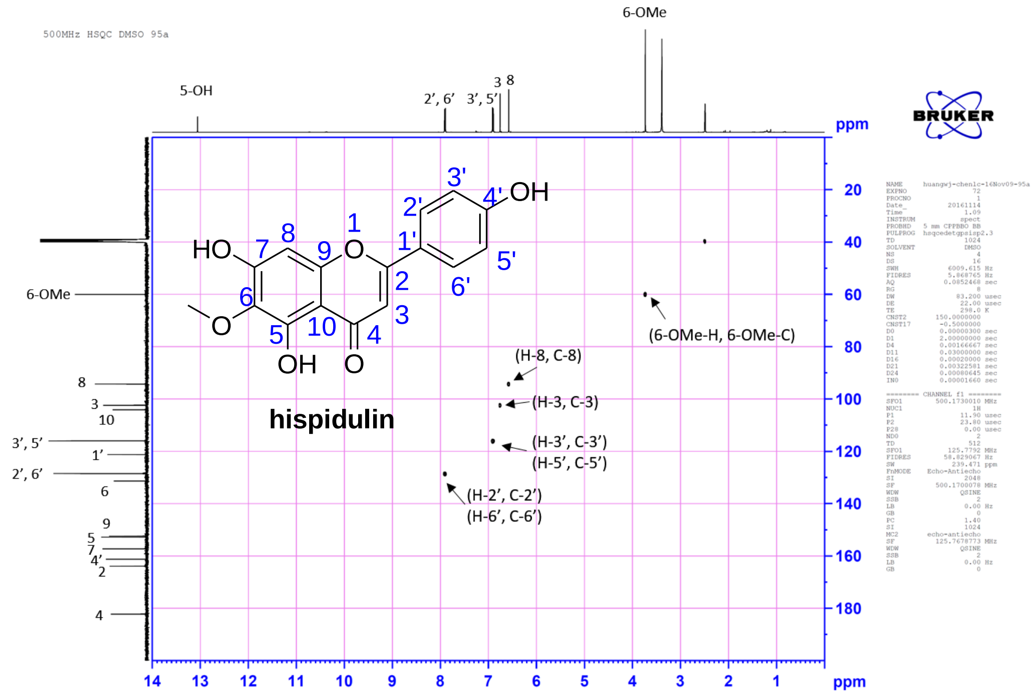

Figure S25. HSQC spectrum of hispidulin

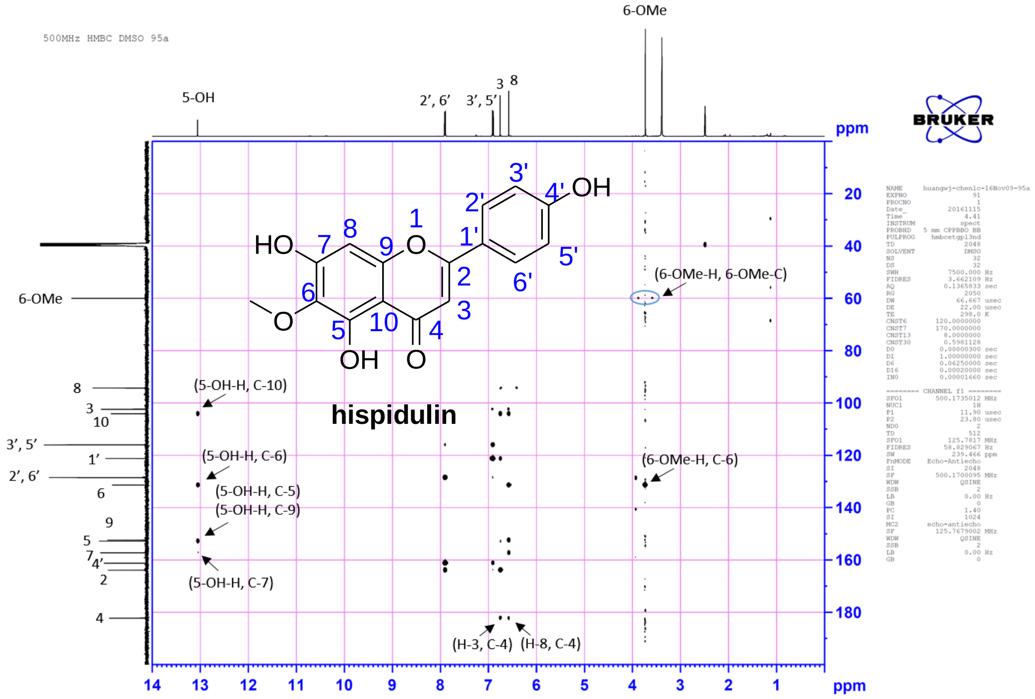

Figure S26. HMBC spectrum of hispidulin

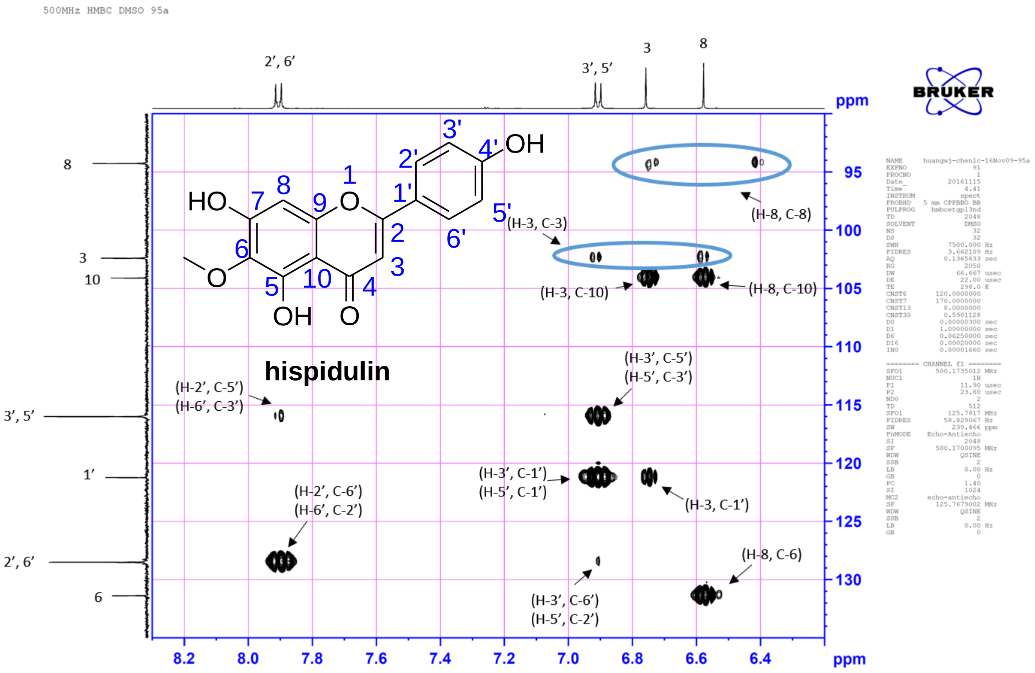

Figure S27. HMBC spectrum of hispidulin

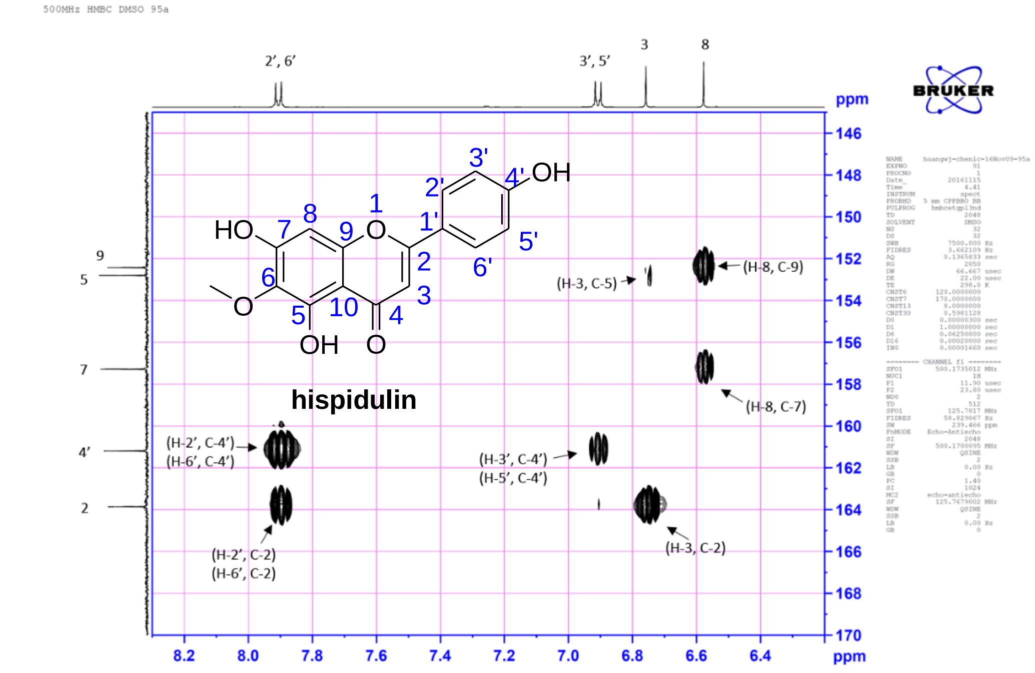

Figure S28. HMBC spectrum of hispidulin

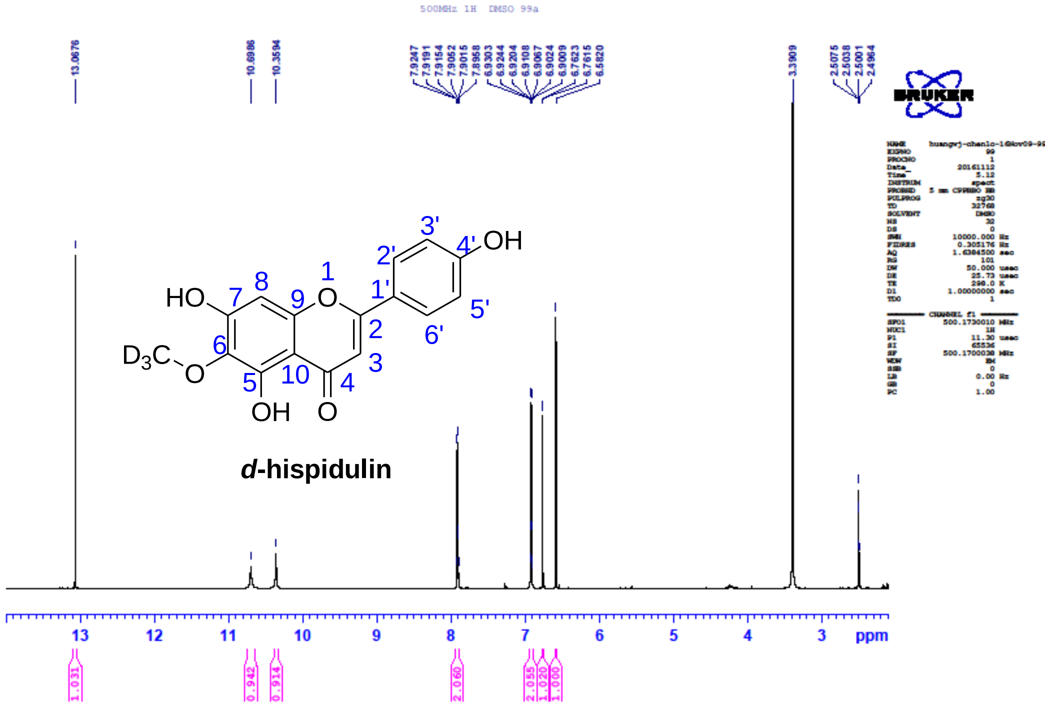

Figure S29. ^{1}H NMR (DMSO-d_6, 500 MHz) spectrum of *d*-hispidulin

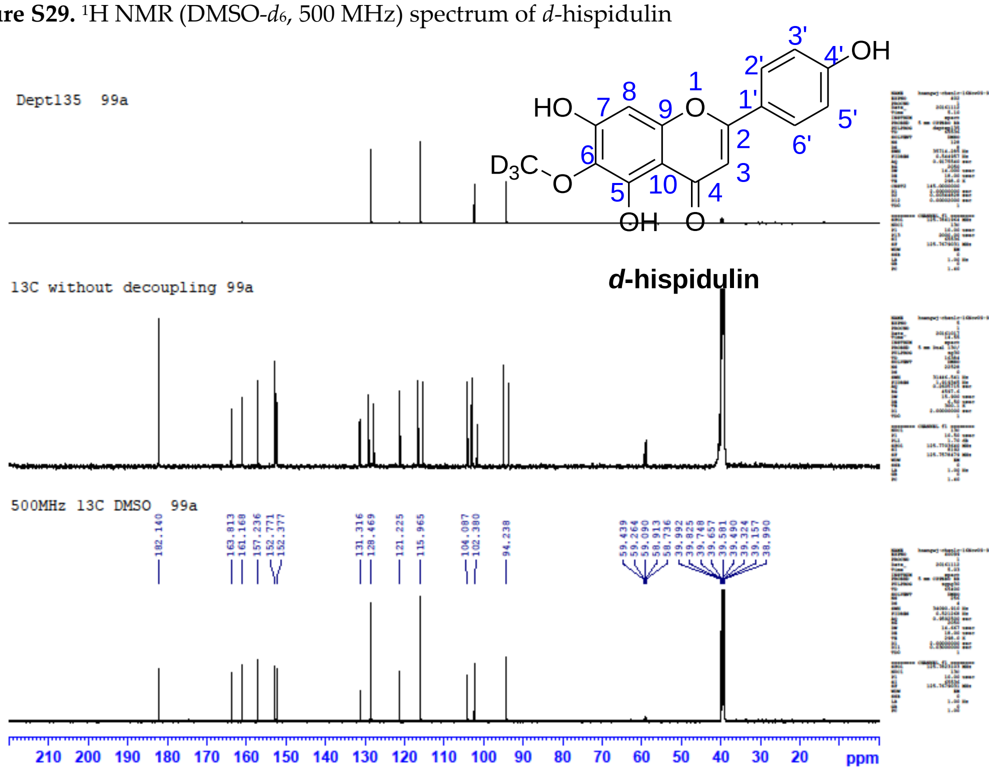

Figure S30. ^{13}C NMR (DMSO-d_6, 125 MHz) spectrum of *d*-hispidulin

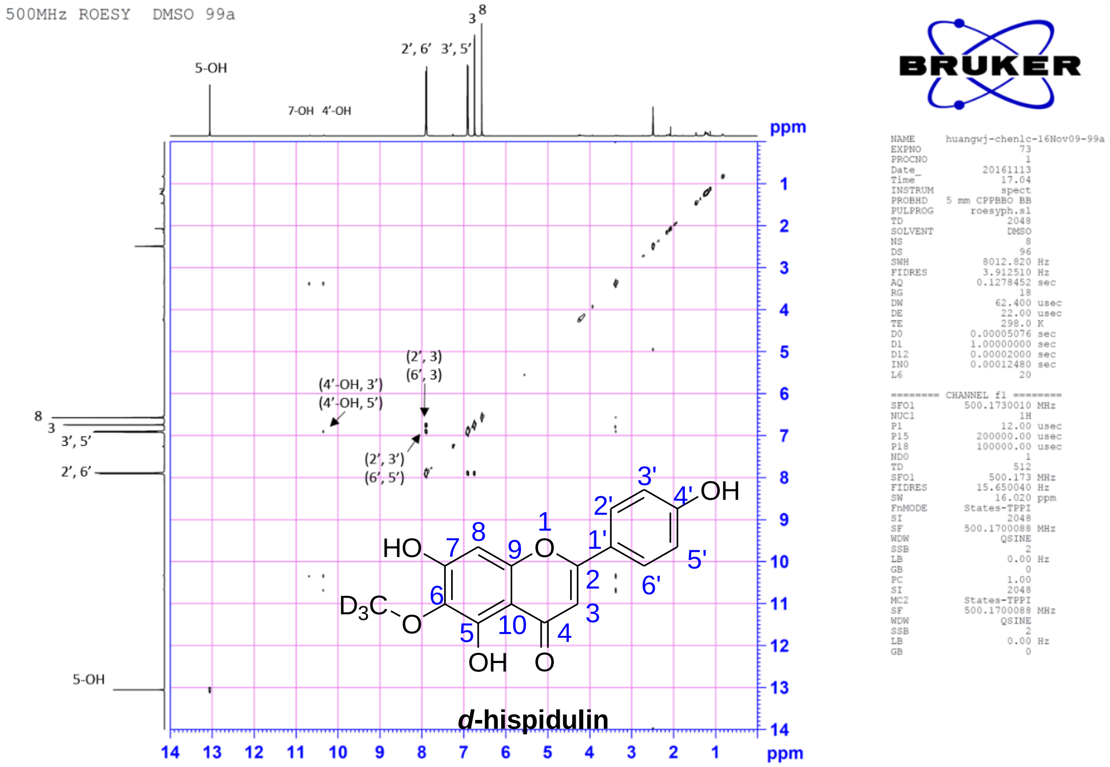

Figure S31. ROESY spectrum of *d*-hispidulin

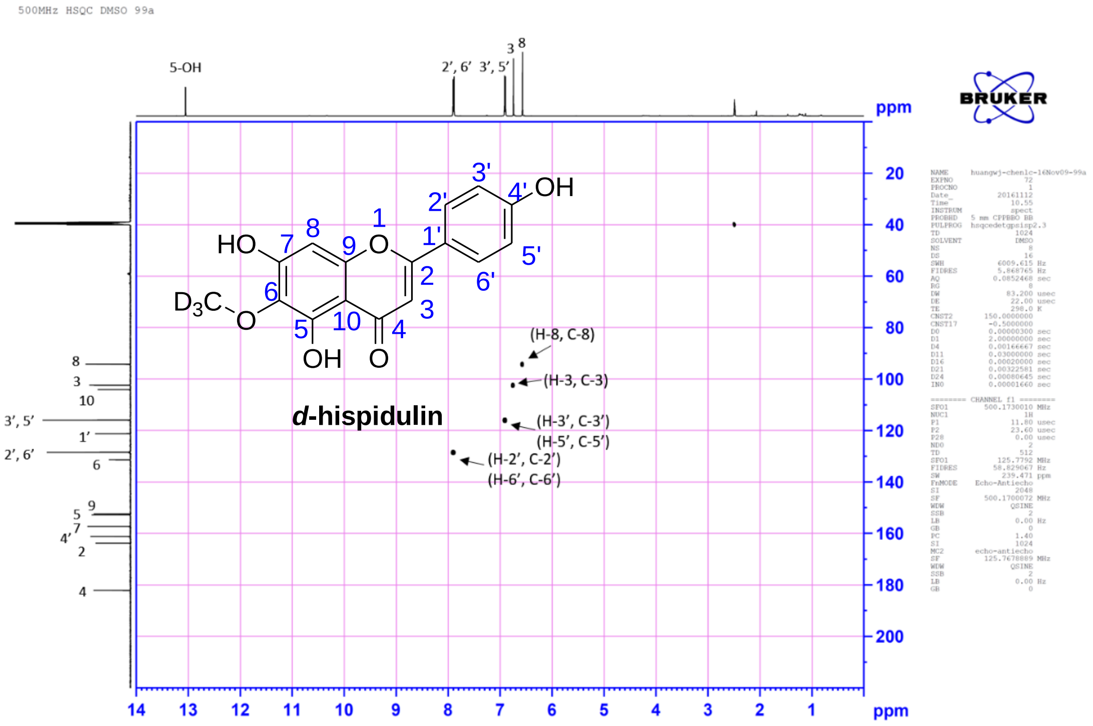

Figure S32. HSQC spectrum of *d*-hispidulin

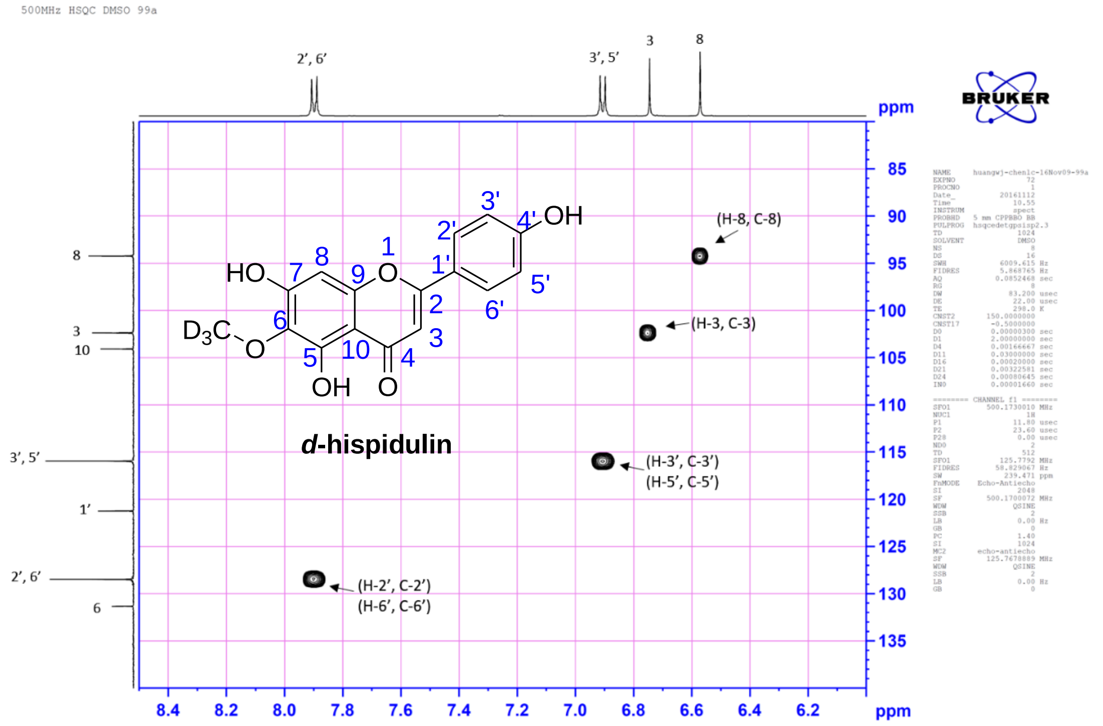

Figure S33. HSQC spectrum of *d*-hispidulin

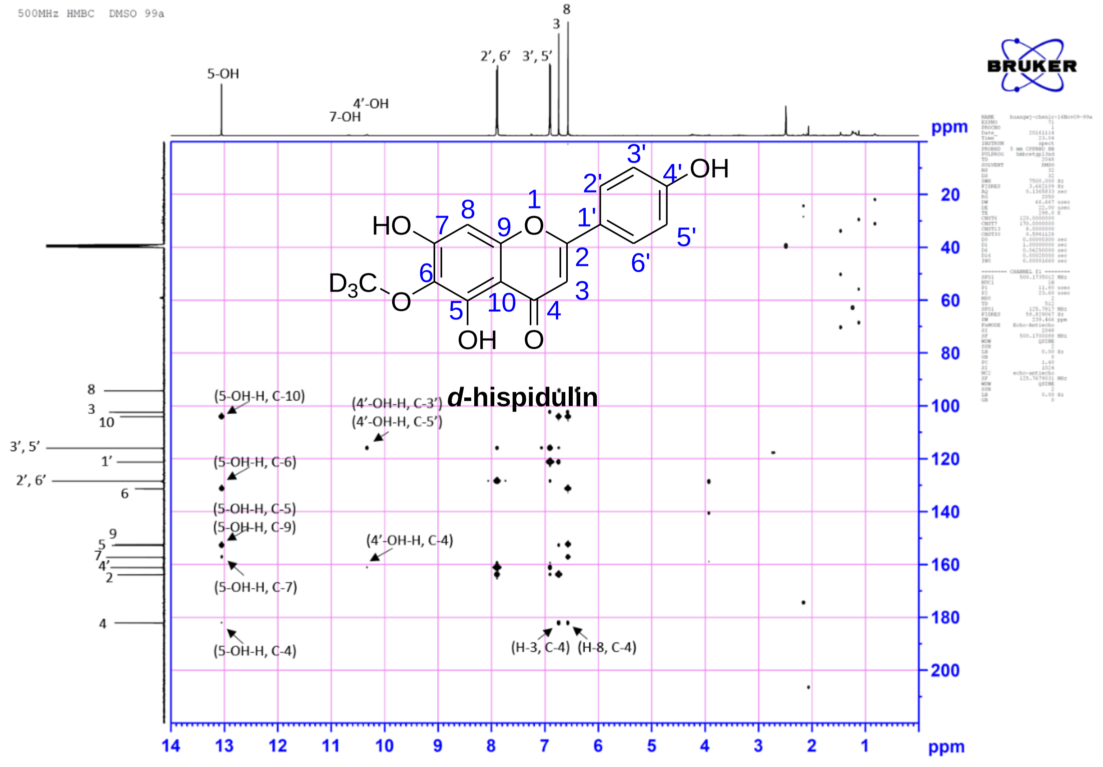

Figure S34. HMBC spectrum of *d*-hispidulin

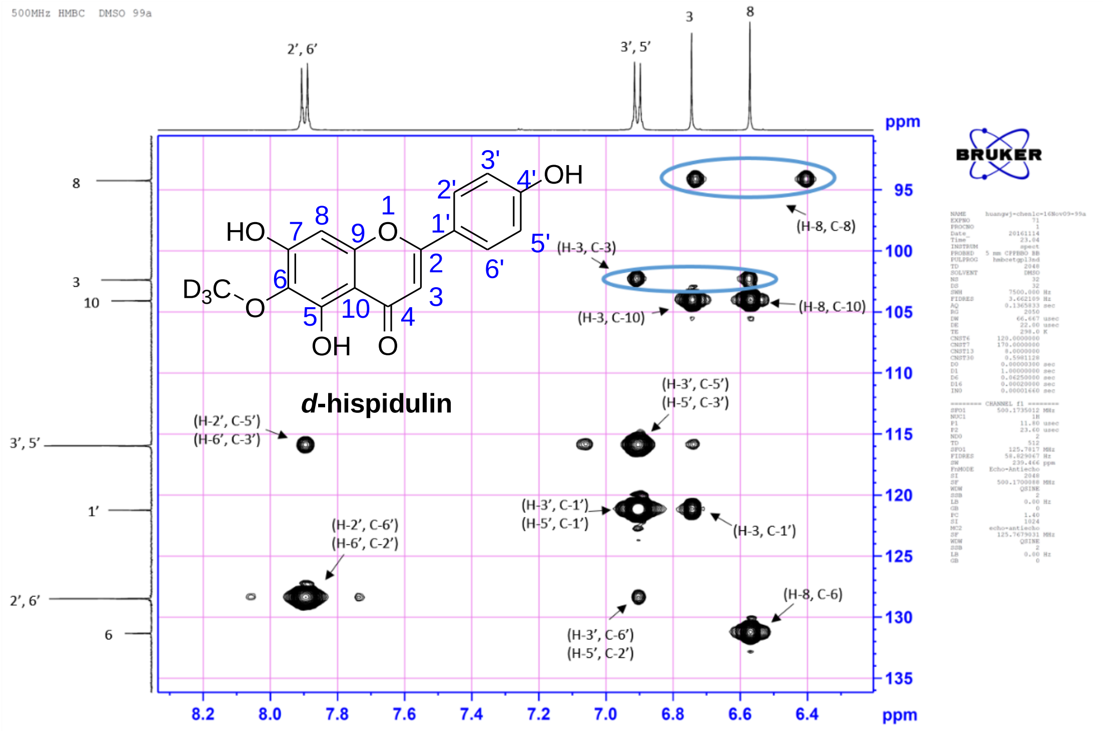

Figure S35. HMBC spectrum of *d*-hispidulin

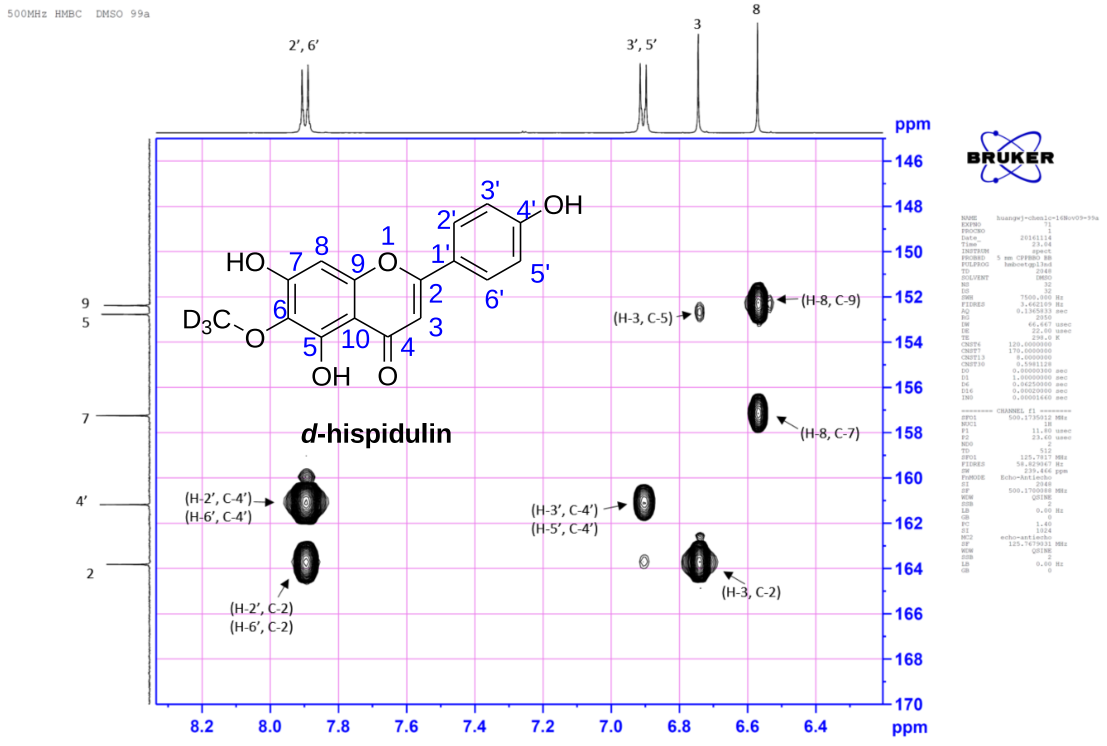

Figure S36. HMBC spectrum of *d*-hispidulin

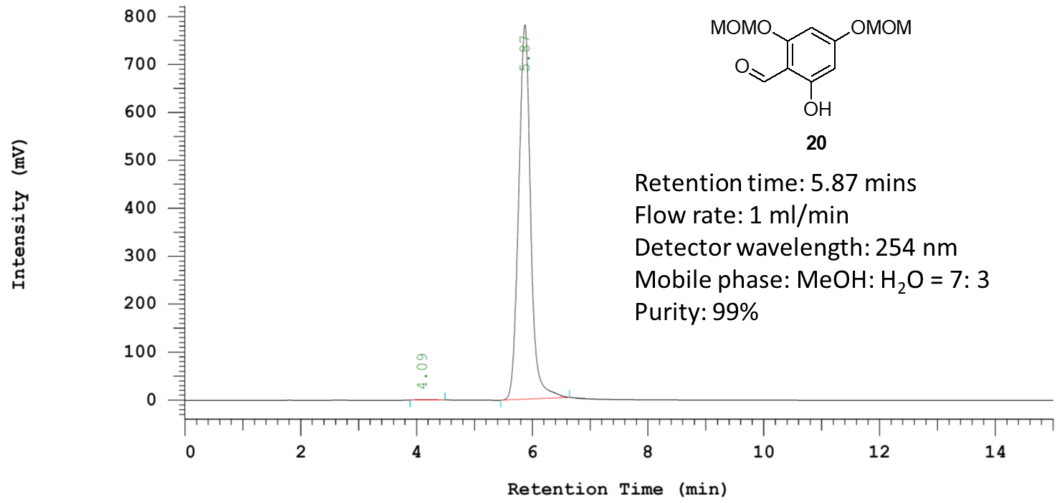

Figure S37. HPLC chromatogram of compound **20**

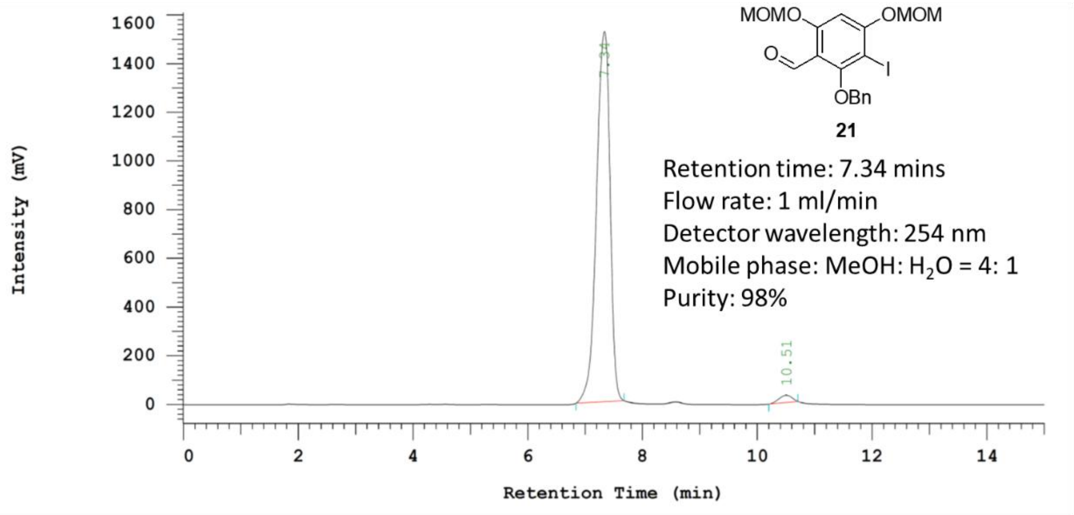

Figure S38. HPLC chromatogram of compound **21**

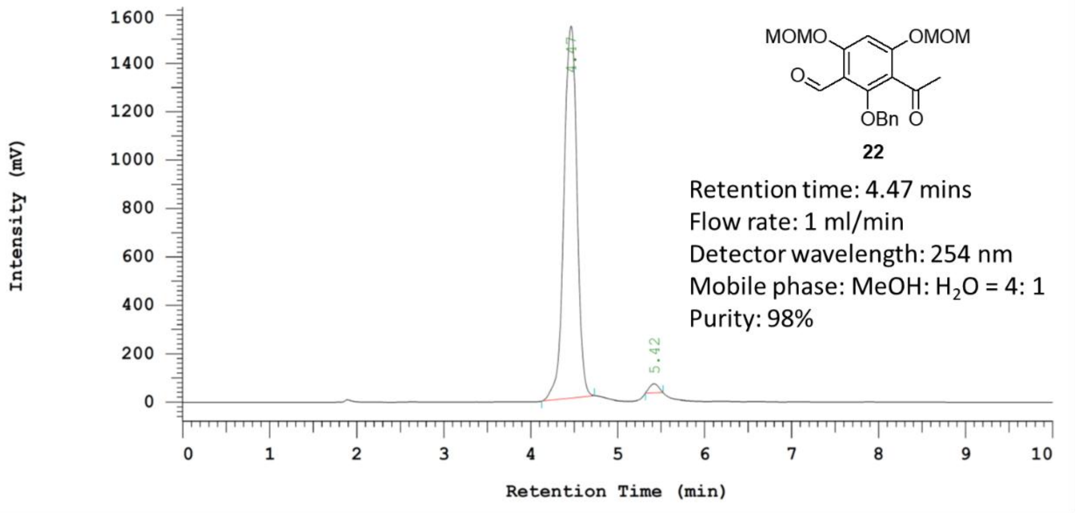

Figure S39. HPLC chromatogram of compound **22**

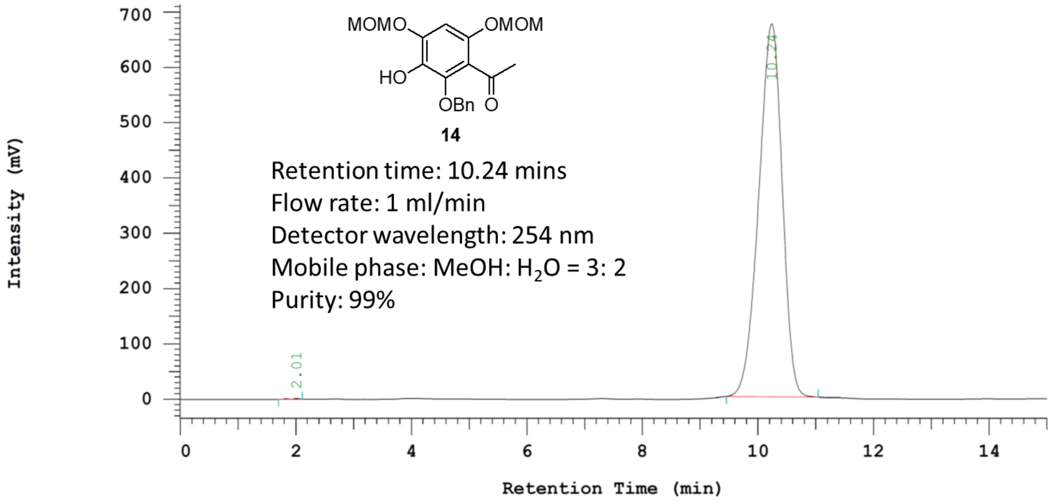

Figure S40. HPLC chromatogram of compound **14**

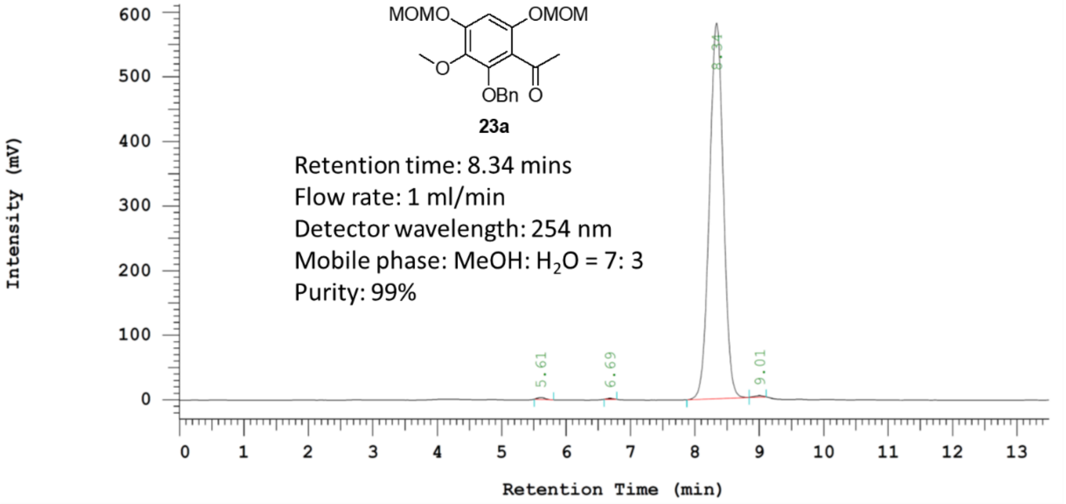

Figure S41. HPLC chromatogram of compound **23a**

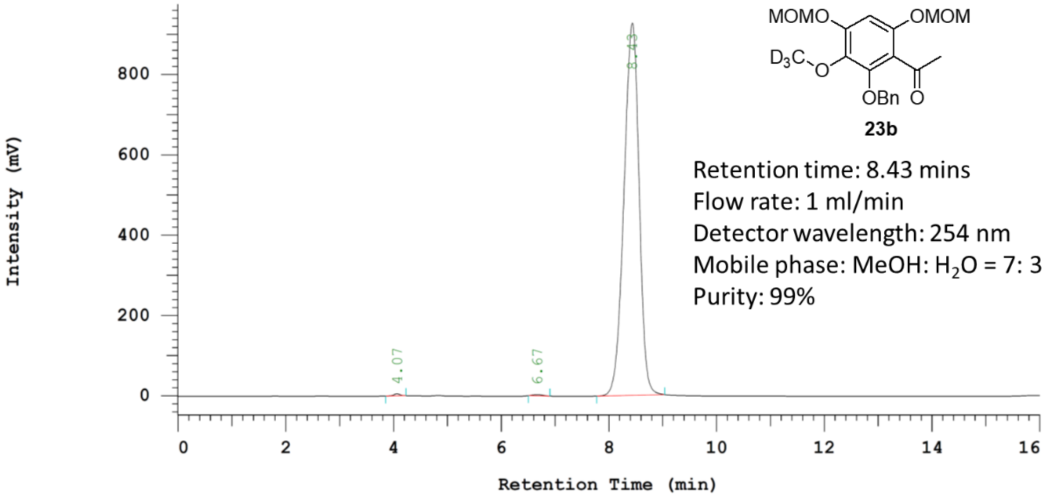

Figure S42. HPLC chromatogram of compound **23b**

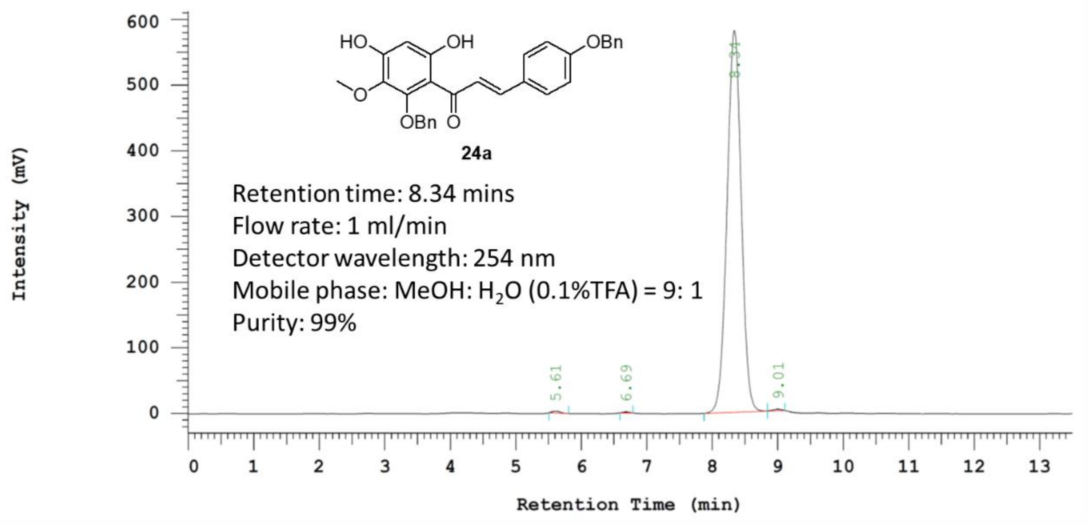

Figure S43. HPLC chromatogram of compound **24a**

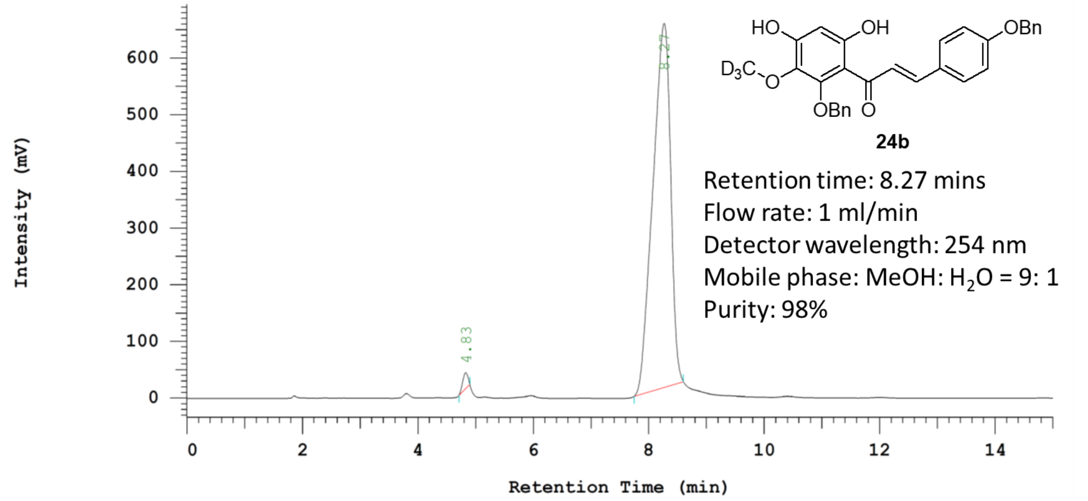

Figure S44. HPLC chromatogram of compound **24b**

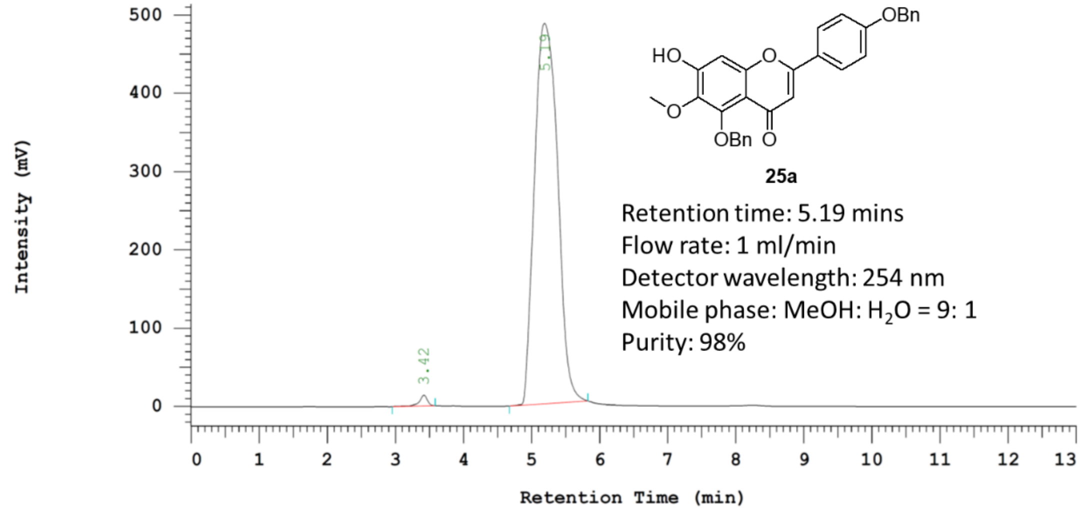

Figure S45. HPLC chromatogram of compound **25a**

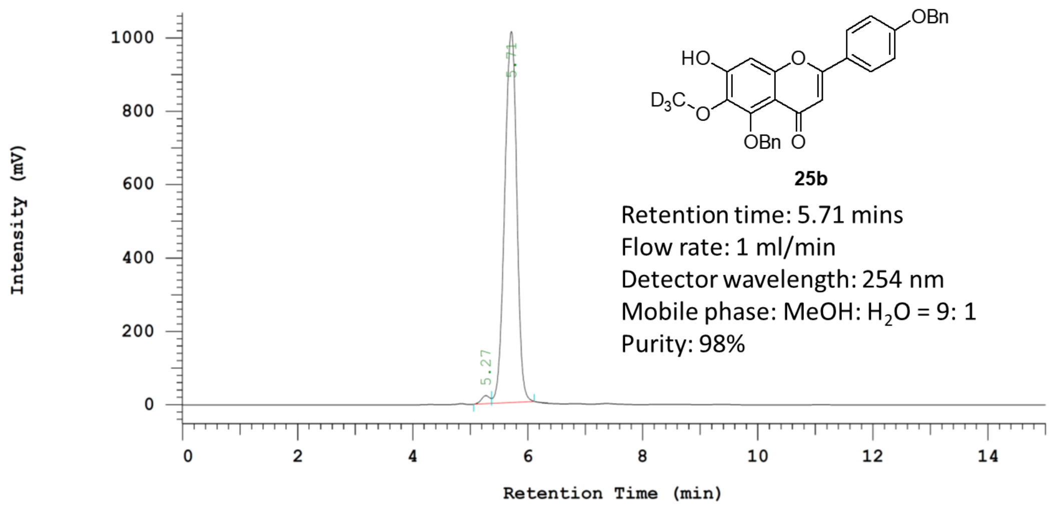

Figure S46. HPLC chromatogram of compound **25b**

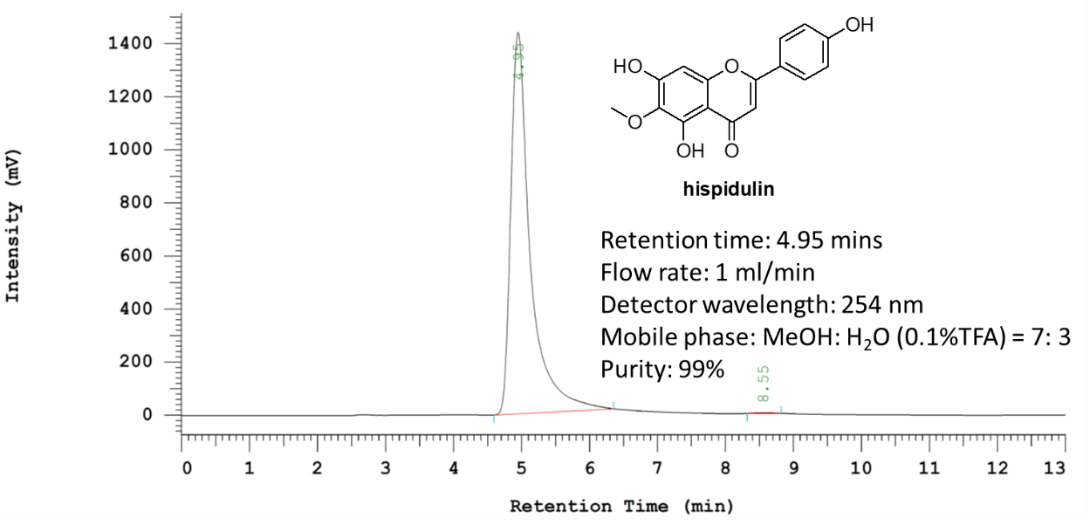

Figure S47. HPLC chromatogram of hispidulin

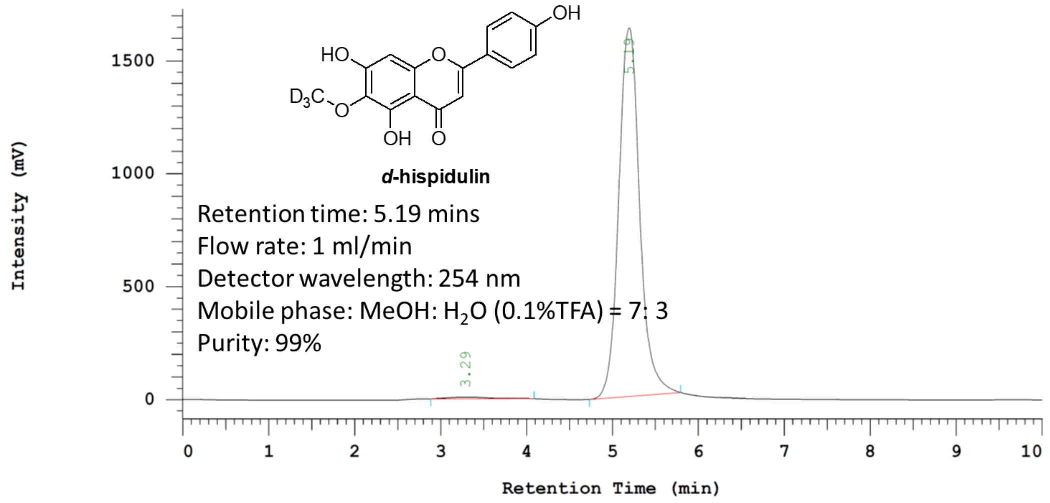

Figure S48. HPLC chromatogram of *d*-hispidulin
